# Simultaneous profiling of sexually transmitted bacterial pathogens, microbiome, and concordant host response in cervical samples using whole transcriptome sequencing analysis

**DOI:** 10.15698/mic2019.03.672

**Published:** 2019-01-24

**Authors:** Catherine M. O'Connell, Hayden Brochu, Jenna Girardi, Erin Harrell, Aiden Jones, Toni Darville, Arlene C. Seña, Xinxia Peng

**Affiliations:** 1 Department of Pediatrics, University of North Carolina at Chapel Hill, Chapel Hill, North Carolina, USA.; 2 Department of Molecular Biomedical Sciences, North Carolina State University, Raleigh, North Carolina, USA.; 3 Department of Medicine, University of North Carolina at Chapel Hill, Chapel Hill, North Carolina, USA.; 4 Bioinformatics Research Center, North Carolina State University, Raleigh, North Carolina, USA.

**Keywords:** RNA-seq, chlamydia, gonorrhea, natural infection, microbiome

## Abstract

Pelvic inflammatory disease (PID) is a female upper genital tract inflammatory disorder that arises after sexually transmitted bacterial infections (STI). Factors modulating risk for reproductive sequelae include co-infection, microbiota, host genetics and physiology. In a pilot study of cervical samples obtained from women at high risk for STIs, we examined the potential for unbiased characterization of host, pathogen and microbiome interactions using whole transcriptome sequencing analysis of ribosomal RNA-depleted total RNAs (Total RNA-Seq). Only samples from women with STI infection contained pathogen-specific sequences (3 to 38% transcriptome coverage). Simultaneously, we identified and quantified their active microbial communities. After integration with host-derived reads from the same data, we detected clustering of host transcriptional profiles that reflected microbiome differences and STI infection. Together, our study suggests that total RNA profiling will advance understanding of the interplay of pathogen, host and microbiota during natural infection and may reveal novel, outcome-relevant biomarkers.

## INTRODUCTION

Pelvic inflammatory disease (PID) continues to pose great risk to the reproductive health of women worldwide [1, 2]. In addition to the cost of treating acute illness, its potentially devastating long-term sequelae can persist throughout a woman's reproductive years. PID occurs when sexually transmitted infections (STIs) ascend from the cervix to the uterus and oviducts, resulting in endometritis and salpingitis. Infection with *Chlamydia trachomatis* (CT), *Neisseria gonorrhoeae* (NG), or both, may lead to PID and associated morbidities including infertility, ectopic pregnancy, and chronic pelvic pain. *Mycoplasma genitalium* (MG) has also emerged as a PID-causing pathogen [3-5]. Currently, it is impossible to predict an individual's risk for developing disease with infection so biomarkers that identify women at elevated risk for reproductive morbidities are needed. Transcriptional profiling that compared systemic responses of women with chlamydial PID to women with local cervical infection, identified specific, disease-associated inflammatory pathways [6] and revealed how NG co-infection depressed developing immunity. We also defined a blood-borne, 21 gene biomarker panel that diagnoses endometritis in asymptomatically-infected women with high chlamydial burden [7]. However, our work, and that of others, has exposed knowledge gaps regarding the impact of pathogen burden [8, 9], co-infection [8, 10-12], vaginal microbiota [13, 14] and oral contraceptives [8] on infection outcome and their potential to serve as additional sources of outcome-relevant biomarkers.

In this pilot study, we examined the potential for unbiased characterization of host, pathogen and microbiome interactions at a molecular level and identification of novel, outcome-relevant biomarkers in a single, easily obtained, clinical specimen using whole transcriptome sequencing analysis of ribosomal RNA (rRNA) depleted total RNAs (Total RNA-Seq).

## RESULTS

### Baseline characteristics of participants

A total of ten cervical specimens were available for this pilot study (**Table 1**) from young women with a median age of 22.5 years (range, 19-28 years). Four participants were tested positively for STI pathogens (40%). Two women had NAAT-confirmed CT or NG infection with *Trichomonas vaginalis* (TV) coinfection (**Table 1**). One other participant was diagnosed with TV, and we subsequently detected MG coinfection via qPCR. MG genomic DNA was also detected in one other sample.

**TABLE 1.** Demographic, clinical and microbiological characteristics of pilot samples (N=10).

Patient Identifier	Age	Bacterial STI Pathogen (genome equivalents/swab)			TV Diagnosis^*^
		CT	NG	MG	
1	19	1.08E+05	-	-	positive
2	27	-	-	-	negative
3	28	-	-	-	negative
4	23	-	2.27E+06	-	positive
5	23	-	-	-	negative
6	22	-	-	3.66E+03	positive
7	25	-	-	-	negative
8	21	-	-	2.50E+02	negative
9	21	-	-	-	negative
10	20	-	-	-	negative

* Assessed via wet mount.

- not detected by NAAT and/or qPCR using pathogen-specific primers.

### Simultaneous profiling of STI pathogens, microbiome, and host response using Total RNA-Seq

An average of 26 million +/-2.8 million Total RNA-Seq short read pairs were obtained for each sample (**Table 2**) and assigned as human, bacterial or TV origin after removal of reads corresponding to residual rRNA sequences (0.1-2.5%) that persisted despite rRNA depletion. Distribution of raw counts for each classification varied across samples. We exploited the genome coverage derived from aligning short reads to a large, non-redundant collection of more than 13,000 bacterial genomes, to identify and quantify active microbial communities present in the cervical samples. We noted an inverse correlation between total numbers of bacterial- and human-derived reads, indicating a possible tradeoff between coverage of the human transcriptome and the microbial transcriptomes represented in individual libraries (Supplementary Figure 1A). Improved resolution of human genes was not detected once samples exceeded 10 million human-derived reads, suggesting that human transcriptome coverage was saturated for many of these samples (Supplementary Figure 1B). Sequencing depths obtained for cervico-vaginal microbiota did not appear to correlate with the number of species detected, their diversity or abundance (Supplementary Figure 1C-D) indicating that sample composition influenced measurement of human and microbiome transcriptomes more strongly than sequencing depth for these cervical samples.

**TABLE 2.** Summary of read distribution from cervical RNA libraries after sequencing.

Patient Identifier	Total	rRNA	rRNA (%)	Human	Human (%)	Bacterial	Bacterial (%)	TV	TV (%)
1	24,473,792	332,825	1.36	8,443,636	34.5	4,386,302	17.92	7,804,744	31.89
2	22,111,682	319,791	1.45	4,114,718	18.61	15,215,299	68.81	28,842	0.13
3	31,615,660	752,169	2.38	7,628,174	24.13	16,742,345	52.96	69,082	0.22
4	25,584,080	167,353	0.65	1,271,025	4.97	10,533,772	41.17	1,138,132	4.45
5	26,899,897	633,674	2.36	21,255,809	79.02	3,066,007	11.4	111	<0.01
6	23,333,959	580,004	2.49	11,471,258	49.16	1,090,474	4.67	9,272,575	39.74
7	24,492,681	35,422	0.14	455,956	1.86	13,542,434	55.29	254	<0.01
8	27,012,744	263,825	0.98	14,769,365	54.68	8,165,858	30.23	421	<0.01
9	25,347,522	324,514	1.28	4,319,634	17.04	18,043,328	71.18	7757	0.03
10	29,199,143	415,861	1.42	14,345,092	49.13	6,614,425	22.65	616	<0.01

**Figure 1:**
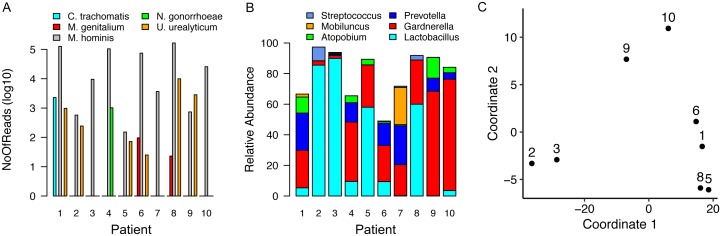
FIGURE 1: Characterization of cervical microbial communities using Total RNA-Seq. **(A)** CT, GC and genital mycoplasma-derived reads detected in cervical RNA libraries. **(B)** Relative abundances of dominant bacterial genera in each sample. **(C)** Clustering of samples based on human gene expression profiles measured in the same samples, visualized by multidimensional scaling. Patient 1 was infected with CT, patients 6 and 8 respectively were infected with MG and are present in the same cluster. Patients 4 (NG) and 7 are not graphed because a low percentage of reads in these samples aligned to the human genome.

Samples obtained from individuals diagnosed with TV via wet mount contained high percentage reads for TV transcripts (4 to 40%) but all remaining samples contained some TV-assigned reads, ranging from <0.01 to 0.22% of the total. Only samples from women with CT, NG or MG infection contained pathogen-specific reads (**Figure 1A**, Supplemental Tables 1-4), with sequences that mapped to common commensals of the human female genital tract such as *M. hominis* and *Ureaplasma urealyticum* [15]. Assigning pathogenspecific reads to annotated genes, a total of 2,285 reads were aligned to 329 protein encoding genes, tRNAs or non-coding RNAs for CT (38.1% transcriptome coverage) (Supplemental Table 1) and 483 reads to 217 GC targets (10.9% coverage) (Supplemental Table 2). For patient samples containing MG, we mapped 60 and 36 reads to 20 and 19 targets respectively (~3.7% coverage/sample). We observed limited overlap, with only *rnpB*, a noncoding RNA and *tufA*, encoding the elongation factor Ef-Tu, common between MG-containing samples (Supplemental Tables 3-4).

To explore interplay between host response, and STI pathogens, we selected the subset of human genes exhibiting the most variable expression (top 500 here) across eight cervical samples for further investigation (Supplementary Table 5). Two samples were excluded because the human-associated reads were insufficient (**Table 2**). Functional enrichment analysis of these 500 selected genes revealed multiple pathways were over-represented (p<0.05) (Supplementary Table 6). Top pathways included HIPPO signaling and epithelial adherens junction signaling, important for epithelial cell health and proliferation. Other pathways included those that contain kalikrein-related peptidases and estrogen receptor signaling, reflecting specific pathways characteristic of cervical epithelium [17]. Interestingly, pathways previously identified as important for establishment of chlamydial infection (Actin Nucleation by ARP-WASP Complex) [18] and subsequent growth (p53 signaling) [19, 20] were also over-represented as were genes involved in granulocyte adhesion and diapedesis, suggesting innate inflammatory signaling activation in some samples.

Recent studies have linked vaginal dysbiosis and elevated bacterial diversity to increased vulnerability to STI such as CT [13, 14] and HIV [21-23]. Here we also observed differences in the microbial relative abundance profiles for each sample (**Figure 1B** and Supplemental Table 7). More interestingly, when eight of these samples were analyzed in the context of the human transcripts also present, we detected clustering that reflected microbiome differences (**Figure 1C**). Samples dominated by *Lactobacillus sp*. clustered away from those in which *Gardnerella* and *Prevotella*, frequently observed with dysbiosis, predominated. The final cluster included samples from women infected with CT or MG. The two samples lacking sufficient human reads, including the specimen obtained from the NG-infected patient, were not assessed (**Table 2**).

## DISCUSSION

Understanding the mechanisms that promote development of genital tract disease and damage after STI infection has proven challenging. CT is an obligate intracellular pathogen with a complex developmental cycle [24] while NG [25] and MG [15] are highly fastidious in their nutritional requirements. Animal models are few [26-28], and large human studies are required to investigate the contribution of host genetic diversity to outcome severity [29]. Polymicrobial communities of anaerobes have been associated with bacterial vaginosis and elevated risk for a variety of reproductive morbidities [30]. In contrast, *Lactobacillus crispatus*-dominated community state types play a protective role against chlamydial infection [13, 14, 31]. It is also possible that uncharacterized PID pathogens hide within the cervicovaginal microbiome [32]. Our pilot study suggests that total RNA profiling can serve as a valuable tool to investigate the interplay of pathogen, host and microbiota during natural infection.

The strengths of this approach are many. Vaginal and cervical swab samples are easily obtained in a clinical setting. The RNA/DNA preservation solution stabilized nucleic acids [33, 34] without need for immediate transfer to -80°C. Quantifying pathogen/co-pathogen abundance provides a route for determining primary drivers of inflammation and outcome. For example, the patient sample with very low levels of MG contained abundant TV transcripts suggesting that the latter was the predominating influence on her innate response. Information regarding gene expression during natural infection by bacterial STI pathogens is lacking [35, 36, 38, 39] but we were pleased to detect expression of between 3-30% of the relevant transcriptome without specific or targeted enrichment. These included pathways critical for central metabolism, surface proteins and non-coding RNAs. Future studies may reveal the influence of host inflammatory or physiologic effects on their regulation and identify novel targets for antimicrobials or suppression of virulence effector expression.

Pathway analysis of the highly variable genes used in clustering host transcriptome profiles of the women in the study revealed significantly enriched pathways reflective of human cervical epithelium. These included pathways important for cell maintenance and proliferation (supplementary data) and pathways that seem of particular importance in the cervical mileu. For example, expression of a family of kalikrein-related peptidases in the epithelial cells of the lower genital tract of women is hormonally regulated [17] to control desquamation and remodeling of cervical mucus. Some members of this family may also contribute to innate defenses by processing/activating antimicrobial α and defensin-1β proteins in mucus [17]. Chlamydial infection may impact epithelial cell health directly by downregulating adherens junction molecule nectin-1 [37], disrupting epithelial tissue homeostasis [38] and inducing epithelialmesenchyme transition [39] and we observed that these pathways, as well as pathways promoting chlamydial entry into cells [18] and subsequent growth [19, 20] were over-represented amongst the most dynamic human transcripts in our small sample population. The impact of MG infection on cervical epithelial responses has yet to be investigated *in vivo*, but *in vitro* chronic infection of human cervical epithelial cells elicits inflammatory cytokines [40, 41]. These very preliminary observations suggest that future studies exploiting Total RNA-Seq have the potential to provide unique insights into dynamic host-pathogen interactions at this infection portal.

Integrating transcriptional data from pathogen(s), microbiota and local host response revealed differential hostmicrobiome relationships between women with *Lactobacillus sp*. versus those with *Gardnerella*/*Prevotella*. Furthermore, women with STI infections clustered separately, possibly reflecting local pathogen-specific inflammatory responses and paralleling systemic responses we have detected in blood [6]. Recent advances in analysis of high-dimensional data will assist interpretation of these observations in the context of carefully designed studies with sufficient power to reveal and validate key pathways and interactions that drive tissue damage and poor reproductive outcomes.

This pilot study also identified potential challenges analyzing clinical specimens. Infection severity is likely to influence representation of pathogen-derived transcripts. We did not have enough samples to compare the impact of lower or higher levels of STI infection on transcriptome coverage. Over-representation of TV transcripts as a result of high levels of co-infection might have also suppressed detection of pathogen sequences. It is possible that superior coverage will be obtained from mono-infected samples. Furthermore, in two of ten samples we did not recover sufficient host-derived material to be able to assess this compartment. Cytobrushes may improve collection of sufficient host material for extraction and analysis [42-44]. Depth of coverage for pathogen genes was low. Improving sensitivity may require a large number of samples for aggregation via bioinformatic analysis or specific, targeted enrichment of pathogen-derived transcripts [45-47]. High sequence conservation of genes encoding core microbial metabolic functions may introduce mis-assignment of pathogen- or microbiome-derived reads for individual genes. Our analysis is also likely biased toward currently available bacterial genomes that may not reflect the full diversity of genital tract microbiota. Future studies optimizing key technical procedures and with increased sample size can address or mitigate many of these challenges, to offer improved understanding of the interplay between STI pathogens, local microbial communities and host responses in development of genital tract disease. Such information will accelerate identification of biomarkers that identify women with subclinical STI-induced reproductive morbidities for more intensive screening or for evaluation of novel therapeutics or vaccines.

## MATERIALS AND METHODS

### Study population

Symptomatic women between the ages of 18-29 years of age who presented for routine evaluation to the public STI clinic located in Durham, North Carolina, were recruited for the study. Sole exclusion criterion was use of antimicrobials in the four weeks prior to sampling. The Institutional Review Board for Human Subject Research at the University of North Carolina approved the study (#17-2175), and all participants provided written informed consent prior to sample collection. Vaginal and cervical specimens were collected from female participants during pelvic examinations in an ongoing pilot study intended to develop a new diagnostic/prognostic clinical tool for STIs. Participants were tested for TV by wet mount microscopy [48] and CT and NG using nucleic acid amplification tests, (Aptima Combo 2; Hologic, Marlborough, MA).

### Sample collection and processing

Cervical swab specimens were collected and stored at -80°C in tubes containing zircon beads and preservative solution (RNA/DNA Shield, Zymo Research, Irvine, CA). Thawed samples were agitated vigorously using a FastPrep Beadbeater (MP Biomedicals, Santa Ana, CA) to release adherent material then DNA and total RNA were simultaneously extracted using a Quick DNA/RNA™ nucleic acid isolation kit (Zymo Research) with on-column DNAse I treatment of the RNA.

### Pathogen quantitation by qPCR

Cervical pathogen burden (CT and MG) for infected study participants was estimated via quantitative PCR [49, 50] using the genomic DNA extracted from swab eluates as template. Similarly, primers directed toward a conserved locus [51] (VT05_017777 F 5' GTCTCGGACAGTCATTCCTCA 3’ and VT05_017777 R 5' CGGAGCAAGACCAAACAGAA 3') were used to quantify NG load.

### Library prep and sequencing

500 ng total RNA was depleted using Illumina Ribo-Zero Epidemiology kit. Libraries were prepared using KAPA RNA Hyper-Prep Kit with RiboErase (HMR) (12 cycles PCR amplification) and dual indexed with a KAPA Dual-Indexed Adapter Kits (1.5µM final concentration). Libraries were sequenced using a Nextseq High-Output Kit utilizing 76bp paired end reads.

### Data processing

Raw reads were demultiplexed and quality trimmed using the standard Illumina bcl2fastq conversion software. Reads were aligned to a custom human rRNA index and then to the hg38 genome with GENCODE v25 primary assembly annotation using STAR v2.5.2b in quantmode [52]. The remaining unmapped reads were then aligned using Bowtie2 v2.3.4 [53] to a pre-built index consisting of over 13,000 bacterial whole genome reference sequences [54]. SLIMM v0.2.2 was used to assign uniquely mapped reads using species-level taxonomic annotation [54]. HTSeq count v0.9.1 was used to generate gene counts for bacterial species of interest, namely CT, NG, and MG [55]. Finally, the leftover reads that did not map to the collection of bacterial genomes were aligned to the TV reference genome using Bowtie2 v2.3.4.

### MDS and IPA pathway analysis

Multidimensional scaling (MDS) analysis was performed using human gene read counts. Counts were normalized and log transformed using DESeq2 [56] and the top 500 variable genes were selected based on their variance across the eight samples included in the analysis. Two samples (4 and 7) were excluded due to insufficient human expression. Functional enrichment analysis of these 500 genes was conducted using Ingenuity Pathway Analysis (QIAGEN Inc., https://www.qiagenbioinformatics.com/products/ingenuity-pathway-analysis), which reported pathways detected and assessed whether they were significantly over-represented using right-tailed Fisher Exact Tests.

All sequence data arising from this study has been deposited into NCBI's GEO under accession GSE120192.

## SUPPLEMENTAL MATERIAL

Click here for supplemental data file.

All supplemental data for this article are available online at http://www.microbialcell.com/researcharticles/2019a-o'connell-microbial-cell/.

## Abbreviations:

CT: Chlamydia trichomatis,
MG: Mycoplasma genitalium,
NG: Neisseria gonorrhoeae,
PID: pelvic inflammatory disease,
rRNA: ribosomal RNA,
STI: sexually transmitted infection,
TV: Trichomonas vaginalis.
