# Supplementary material for: Simultaneous profiling of sexually transmitted bacterial pathogens, microbiome, and concordant host response in cervical samples using whole transcriptome sequencing analysis
Source: Microb Cell. 2019 Jan 24;6(3):177–83. doi: 10.15698/mic2019.03.672 (PMC6402362; doi:10.15698/mic2019.03.672)
Supplement: Supplementary file 1 [file mic-06-177-s01.pdf]

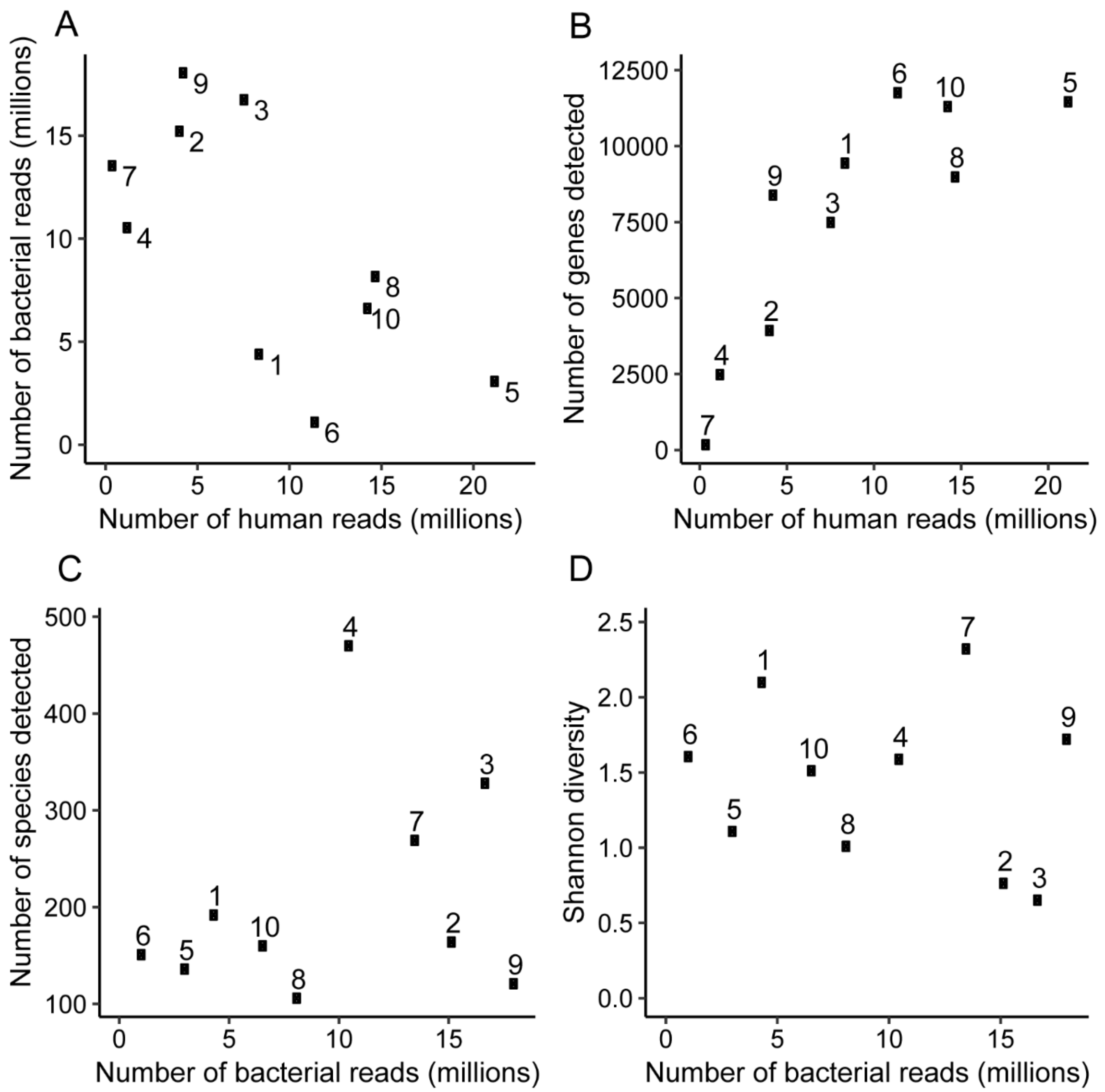

Supplementary Figure 1. Scatterplots showing the assignment of Total RNA-seq reads and measures of human genes and bacterial species detected in each sample. (A) Bacterial versus human-derived reads (millions). (B) Number of genes detected (minimum 20 assigned reads) versus human-derived reads (millions). (C) Number of bacterial species (minimum 20 assigned reads) versus bacterial-derived reads (millions). (D) Shannon diversity versus the number of bacterial-derived reads (millions).

Supplementary Table 1. Patient #1 CT alignment

CT gene	CT protein	SLIMM_all	SLIMM_unique
ompA	Major Outer Membrane Protein	211	211
tmRNA	noncoding RNA	173	173
omcA	9kDa-Cysteine-Rich Lipoprotein	100	100
omcB	60kDa Cysteine-Rich OMP	66	66
CT_398.1	noncoding RNA	61	61
groEL_1	HSP-60	39	39
ndk	Nucleoside-2-P Kinase	33	33
CT_858	predicted Protease containing IRBP and DHR domains	29	29
tufA	Elongation Factor Tu	28	28
hctB	Histone-like Protein 2	24	24
yscC	probable Yop proteins translocation protein C/general secretion pathway protein	23	23
CT_081	CHLTR T2 Protein	26	20
rpoC	RNA Polymerase Beta'	24	20
CT_671	hypothetical protein	20	20
clpC	ClpC Protease ATPase	19	19
CT_578	hypothetical protein	19	18
CT_664	adenylate cyclase-like protein	18	17
rho	Transcription Termination Factor	15	15
glgA	Glycogen Synthase	15	15
CT_456	hypothetical protein	14	14
CT_875	hypothetical protein	14	14
CT_708	SWF/SNF family helicase	13	13
dhnA	Predicted 1%2C6-Fructose biphosphate aldolase (dehydrin family)	12	12
clpX	CLP Protease ATPase	15	12
CT_082	hypothetical protein	13	11
nusA	Transcription antitermination factor	11	11
CT_266	hypothetical protein	11	11
ihfA	Integration Host Factor Alpha	11	11
tal	Transaldolase	11	11
fusA	Elongation Factor G	11	11
CT_579	hypothetical protein	12	11
recA	hypothetical protein	10	10
CT_043	hypothetical protein	9	9
rl1	L1 Ribosomal Protein	11	9
yprS	hypothetical protein	9	9
pmpC	Putative outer membrane protein C	9	9
secY	Translocase	9	9
lcrH_1	Low Calcium Response Protein H	10	9
ung	Uracil DNA Glycosylase	9	9
CT_610	hypothetical protein	10	9
pyrH	UMP Kinase	10	9
ftsW	Cell Division Protein FtsW	9	9
nrdA	Ribonucleoside Reductase%2C Large Chain	9	9
pmpE	Putative Outer Membrane Protein E	12	9
pmpH	Putative Outer Membrane Protein H	9	9
CT_001	hypothetical protein	8	8
gyrA_1	DNA Gyrase Subunit A	8	8
pmpB	Putative outer membrane protein B	8	8
rs5	S5 Ribosomal Protein	8	8
dnaE	DNA Pol III Alpha	8	8
CT_546	predicted OMP	8	8
porB	Outer Membrane Protein Analog	8	8
tktB	Transketolase	8	8
pmpD	Putative Outer Membrane Protein D	8	8
glgB	1%2C 4-alpha-glucan branching enzyme	8	8
pmpF	Putative Outer Membrane Protein F	11	8
lcrD	Low Calcium Response D	10	7
trxB	Thioredoxin Reductase	7	7
CT_214	hypothetical protein	8	7
CT_289	hypothetical protein	7	7

gidA	FAD-dependent oxidoreductase	7	7
gapA	Glyceraldehyde-3-P Dehydrogenase	7	7
rl3	L3 Ribosomal Protein	11	7
CT_556	hypothetical protein	7	7
CT_622	CHLPN 76kDa Homolog	7	7
yceA	hypothetical protein	7	7
CT_663	hypothetical protein	7	7
fabF	Acyl Carrier Protein Synthase	9	7
htrA	DO Serine Protease	7	7
pheS	Phenylalanyl tRNA Synthetase%2C Alpha	7	7
pmpI	Putative Outer Membrane Protein I	7	7
dcd	Deoxycytidine triphosphate deaminase family protein	6	6
ytgC	Integral Membrane Protein	6	6
CT_115	hypothetical protein	6	6
CT_136	predicted Lysophospholipase esterase	6	6
yaeT	Omp85 Analog	6	6
CT_283	hypothetical protein	6	6
rpoB	RNA Polymerase Beta	10	6
CT_503	hypothetical protein	6	6
rl6	L6 Ribosomal Protein	6	6
rl2	L2 Ribosomal Protein	6	6
uhpC	Hexosphosphate Transport	6	6
lpdA	Lipoamide Dehydrogenase	6	6
CT_560	hypothetical protein	7	6
CT_565	hypothetical protein	6	6
CT_621	hypothetical protein	6	6
CT_623	CHLPN 76kDa Homolog	6	6
CT_665	hypothetical protein	10	6
CT_668	hypothetical protein	7	6
pgk	Phosphoglycerate Kinase	6	6
CT_814	hypothetical protein	7	6
ftsY	Cell Division Protein FtsY	6	6
nrdB	Ribonucleoside Reductase%2C Small Chain	6	6
pmpG	Putative Outer Membrane Protein G	6	6
gatB	Glu-tRNA Gln Amidotransferase B Subunit	7	5
ssb	SS DNA Binding Protein	5	5
CT_065	ADP/ATP Translocase	5	5
clpB	Clp Protease ATPase	6	5
CT_365	hypothetical protein	5	5
mdhC	Malate Dehydrogenase	5	5
gltX	Glutamyl-tRNA Synthetase	6	5
CT_700	hypothetical protein	5	5
rs18	S18 Ribosomal Protein	5	5
rl35	L35 Ribosomal Protein	5	5
CT_837	hypothetical protein	6	5
ftsH	ATP-dependent zinc protease	5	5
lepA	GTPase	4	4
ytgA	Solute Protein Binding Family	4	4
ltuB	hypothetical protein	8	4
CT_181	hypothetical protein	4	4
accD	AcCoA Carboxylase/Transferase Beta	4	4
sodM	Superoxide Dismutase (Mn)	4	4
lon	Lon ATP-dependent protease	4	4
def	Polypeptide Deformylase	4	4
CT_388	hypothetical protein	4	4
yrbH	GutQ/KpsF Family Sugar-P Isomerase	4	4
rs7	S7 Ribosomal Protein	4	4
tsp	Tail-Specific Protease	4	4
recJ	ssDNA Exonuclease	4	4
cdsA	Phosphatidate Cytidylytransferase	4	4
oppA_4	oligopeptide Binding Lipoprotein	4	4
fliY	Glutamine Binding Protein	4	4

CT_504	hypothetical protein	4	4
lpxC	UDP-3-O-Acyl GlcNAc Deacetylase	4	4
gspD	Gen. Secretion Protein D	4	4
folP	Dihydropteroate Synthase	4	4
pkn5	S/T Protein Kinase	4	4
tsf	Elongation Factor TS	8	4
CT_685	ABC Transporter ATPase	4	4
CT_694	hypothetical protein	4	4
mreB	Rod Shape Protein-Sugar Kinase	4	4
pgm	Phosphoglycerate Mutase	4	4
mfd	Transcription-Repair Coupling	4	4
murD	UDP-N-acetylmuramoylalanine-D-glutamate ligase	7	4
ldh	Leucine Dehydrogenase	4	4
aas	Acylglycerophosphoethanolamine Acyltransferase	4	4
CT_790	hypothetical protein	4	4
yccA	Transport Permease	4	4
xerD	Integrase/recombinase	4	4
CT_016	hypothetical protein	3	3
sucB_1	Dihydrolipoamide Succinyltransferase	3	3
lcrE	Low Calcium Response E	3	3
groES	10KDa Chaperonin	3	3
zwf	Glucose-6-P Dehyrogenase	3	3
gyrB_1	DNA Gyrase Subunit B	4	3
gseA	KDO Transferase	3	3
murE	UDP-N-acetylmuramoylalanylglutamyl DAP Ligase	3	3
mrsA_1	Phosphomannomutase	3	3
rl7	L7/L12 Ribosomal Protein	3	3
rl11	L11 Ribosomal Protein	5	3
lysC	Aspartokinase III	4	3
CT_372	hypothetical protein	3	3
pgi	Glucose-6-P Isomerase	3	3
dnaK	HSP-70	3	3
lpxK	probable tetraacyldisaccharide 4' kinase	3	3
CT_444.1	hypothetical protein	4	3
CT_460	SWIB (YM74) complex protein	3	3
yaeI	phosphohydrolase	3	3
rl4	L4 Ribosomal Protein	7	3
yciA	Acyl-CoA Thioester Hydrolase	3	3
mip	FKBP-type peptidyl-prolyl cis-trans isomerase	3	3
yscL	Yop proteins translocation protein L	3	3
CT_567	hypothetical protein	4	3
CT_577	hypothetical protein	4	3
tolB	component of a macromolecule transport system	3	3
ahpC	Thio-specific Antioxidant (TSA) Peroxidase	3	3
CT_611	hypothetical protein	4	3
CT_667	hypothetical protein	5	3
fliN	Flagellar Motor Switch Domain/YscQ family	3	3
yfhO_1	NifS-related enzyme	3	3
secA_2	Protein Translocase	3	3
pcnB_2	Poly A Polymerase	6	3
ribD	Riboflavin Deaminase	3	3
CT_734	hypothetical protein	3	3
ftsK	Cell Division Protein FtsK	3	3
hctA	Histone-Like Developmental Protein	3	3
CT_768	hypothetical protein	3	3
CT_775	snGlycerol 3-P Acyltransferase	3	3
tyrP_2	Tyrosine Transport	3	3
CT_824	Zinc Metalloprotease (insulinase family)	3	3
htrB	Acyltransferase	2	2
CT_017	hypothetical protein	2	2
CT_018	hypothetical protein	2	2
ileS	Isoleucyl-tRNA Synthetase	2	2

rnhB_2	Ribonuclease HII	2	2
CT_036	hypothetical protein	2	2
pepA	Leucyl Aminopeptidase A	2	2
CT_049	hypothetical protein	2	2
gcpE	hypothetical protein	2	2
ytgB_1	rRNA methylase	2	2
CT_084	Phopholipase D Superfamily	6	2
malQ	4-alpha glucanotransferase	2	2
yscU	Yop proteins translocation protein U	5	2
efp_1	Elongation Factor P	2	2
rs9	S9 Ribosomal Protein	2	2
CT_138	Dipeptidase	4	2
CT_147	hypothetical protein	2	2
surE	SurE-like Acid Phosphatase	2	2
yqfU	hypothetical protein	7	2
CT_223	hypothetical protein	2	2
incB	Inclusion Membrane Protein B	2	2
fabH	Oxoacyl Carrier Protein Synthase III	2	2
lpxD	UDP-3-O-[3-hydroxymyristoyl] glucosamine N-acyltransferase	2	2
pdhA	Pyruvate Dehydrogenase Alpha	2	2
yhfO	NifS family of pyridoxal phosphate-dependent enzymes	2	2
nqr2	NADH (Ubiquinone) Dehydrogenase	2	2
nqr3	NADH (Ubiquinone) Oxidoreductase%2C Gamma	2	2
CT_309	hypothetical protein	2	2
CT_326.2	hypothetical protein	2	2
xseA	Exodoxyribonuclease VII	2	2
pdhA/B	(pyruvate) Oxoisovalerate Dehydrogenase Alpha/Beta Fusion	2	2
yyaL	multidomain protein family	2	2
CT_358	hypothetical protein	3	2
artJ	Arginine Binding Protein	2	2
CT_384	hypothetical protein	4	2
CT_387	hypothetical protein	2	2
proS	Prolyl tRNA Synthetase	2	2
sucB_2	Dihydrolipoamide Succinyltransferase	2	2
dksA	DnaK Suppressor	5	2
clpP_1	CLP Protease	3	2
rs10	S10 Ribosomal Protein	2	2
rs12	S12 Ribosomal Protein	2	2
yebC	YebC family	2	2
pheT	phenylalanyl tRNA Synthetase Beta	2	2
CT_482	hypothetical protein	2	2
CT_484	hypothetical protein	2	2
pgsA_1	CDP-diacylglycerol--glycerol-3-phosphate 3-phosphatidyltransferase	2	2
rl17	L17 Ribosomal Protein	2	2
rs13	S13 Ribosomal Protein	2	2
rl5	L5 Ribosomal Protein	3	2
rl24	L24 Ribosomal Protein	7	2
rl16	L16 Ribosomal Protein	2	2
CT_529	hypothetical protein	2	2
aspS	Aspartyl tRNA Synthetase	2	2
hisS	Histidyl tRNA Synthetase	2	2
fmu	RNA Methyltransferase	2	2
yscJ	Yop proteins translocation lipoprotein J	3	2
yscT	Yop proteins translocation protein T	2	2
thrS	Threonyl tRNA Synthetase	2	2
minD	chromosome partitioning ATPase-CHLTR plasmid protein homolog GP5D	2	2
uvrB	Exinuclease ABC Subunit B	2	2
pal	Peptidoglycan-Associated Lipoprotein	2	2
uvrD	DNA Helicase	2	2
CT_619	hypothetical protein	2	2
CT_620	hypothetical protein	2	2
CT_635	hypothetical protein	2	2

greA	Transcription Elongation Factor G	2	2
recB	Exodeoxyribonuclease V%2C Beta	2	2
CT_642	hypothetical protein	4	2
CT_652.1	hypothetical protein	2	2
CT_659	hypothetical protein	2	2
gyrB_2	DNA Gyrase Subunit B	2	2
CT_666	hypothetical protein	6	2
yscN	Yops secretion ATPase	2	2
karG	Arginine Kinase	2	2
CT_676	hypothetical protein	2	2
rrf	Ribosome Releasing Factor	2	2
CT_691	hypothetical protein	2	2
CT_702	hypothetical protein	2	2
pckA	Phosphoenolpyruvate Carboxykinase	2	2
CT_715	AgX-1 Homolog-UDP-Glucose Pyrophosphorylase	2	2
zntA	Metal Transport P-type ATPase	2	2
CT_728	hypothetical protein	2	2
CT_733	hypothetical protein	2	2
dagA_2	D-Ala/Gly Permease	2	2
alaS	Alanyl tRNA Synthetase	2	2
efp_2	Elongation Factor P	2	2
nlpD	Muramidase (invasin repeat family)	5	2
murC/ddlA	UDP-N-acetylmuramate-alanine ligase/D-Ala-D-Ala Ligase	3	2
CT_780	Thioredoxin Disulfide Isomerase	2	2
cysS	Cysteinyl tRNA Synthetase	2	2
CT_783	predicted disulfide bond isomerase	3	2
rs14	S14 Ribosomal Protein	5	2
CT_788	leader (60) peptide-periplasmic	3	2
rl9	L9 Ribosomal Protein	2	2
glmS	Glucosamine-Fructose-6-P Aminotransferase	3	2
sucC	Succinyl-CoA Synthetase%2C Beta	2	2
sucD	Succinyl-CoA Synthetase%2C Alpha	2	2
infC	Initiation Factor 3	2	2
tRNAAsp	noncoding RNA	4	2
tRNAThr_2	noncoding RNA	2	2
gatA	Glu-tRNA Gln Amidotransferase (A subunit)	3	1
CT_005	hypothetical protein	1	1
ffh	Signal Recognition Particle GTPase	1	1
CT_035	Biotin Protein Ligase	1	1
CT_041	hypothetical protein	1	1
gnd	6-Phosphogluconate Dehydrogenase	2	1
CT_073	predicted OMP I	2	1
rl28	L28 Ribosomal Protein	1	1
infB	Initiation Factor-2	1	1
CT_134	hypothetical protein	1	1
CT_153	hypothetical protein	1	1
rpiA	Ribose-5-P Isomerase A	2	1
amiA	N-Acetylmuramoyl Alanine Amidase	1	1
atpB	ATP Synthase Subunit B	1	1
yjjK	ABC Transporter Protein ATPase	1	1
CT_352	hypothetical protein	1	1
dapA	Dihydrodipicolinate Synthase	1	1
asd	Aspartate semialdehyde dehydrogenase	2	1
CT_373	hypothetical protein	1	1
CT_386	predicted metal dependent hydrolase	1	1
ribC	Riboflavin Synthase	1	1
dapF	Diaminopimelate Epimerase	2	1
euo	CHLPS Euo Protein	1	1
secD/secF	SecD/SecF fusion protein	1	1
CT_480.1	hypothetical protein	1	1
rs11	S11 Ribosomal Protein	3	1
rs19	S19 Ribosomal Protein	1	1

CT_538	hypothetical protein	1	1
yscR	Yop proteins translocation protein R	1	1
CT_566	hypothetical protein	2	1
rs20	S20 Ribosomal Protein	1	1
CT_618	hypothetical protein	1	1
rs4	S4 Ribosomal Protein	1	1
recC	Exodeoxyribonuclease V%2C Gamma	1	1
ygeD	predicted Efflux Protein	3	1
topA	DNA Topoisomerase I-Fused to SWI Domain	1	1
yohI	predicted oxidoreductase	1	1
kdsA	KDO Synthetase	1	1
gyrA_2	DNA Gyrase Subunit A	1	1
yycJ	metal dependent hydrolase	3	1
dnaG	DNA Primase	1	1
rs6	S6 Ribosomal Protein	1	1
rl32	L32 Ribosomal Protein	1	1
CT_838	hypothetical protein	2	1
CT_849.1	hypothetical protein	1	1
lytB	Metalloprotease	1	1
tRNAThr_3	noncoding RNA	1	1
tyrS	tyrosyl tRNA Synthetase	1	0
yaeL	Metalloprotease	1	0
CT_083	hypothetical protein	4	0
CT_114	hypothetical protein	1	0
oppA_1	Oligopeptide Binding Protein	2	0
CT_191	hypothetical protein	1	0
ubiX	Phenylacrylate Decarboxylase	3	0
CT_357	hypothetical protein	1	0
ycfF	Hit Family Hydrolase	2	0
lspA	Lipoprotein Signal Peptidase	3	0
rl27	L27 Ribosomal Protein	1	0
rl21	L21 Ribosomal Protein	1	0
CT_421.2	hypothetical protein	1	0
CT_422	possible metalloenzyme	1	0
CT_496.1	hypothetical protein	1	0
dnaB	Replicative DNA Helicase	1	0
rpoA	RNA Polymerase Alpha	2	0
rl14	L14 Ribosomal Protein	4	0
rs2	S2 Ribosomal Protein	3	0
CT_737	SET Domain protein	2	0
CT_763	hypothetical protein	1	0
CT_771	hydrolase/phosphatase homolog	2	0
rnpA	Ribonuclease P Protein Component	1	0
rl36	L36 Ribosomal Protein	2	0
CT_814.1	hypothetical protein	1	0
mrsA_2	Phosphoglucomutase	1	0
CT_847	hypothetical protein	1	0
CT_848	hypothetical protein	1	0

Supplementary Table 2. Patient #4 NG alignment

NG gene	NG protein	SLIMM_all	SLIMM_unique
NGO_19101	noncoding RNA	40	40
groEL	molecular chaperone GroEL	18	18
NGO_1577	membrane protein	11	11
aceE	pyruvate dehydrogenase	9	9
rpsA	30S ribosomal protein S1	8	8
NGO_11130	large pilS cassette	8	8
dnaK	molecular chaperone DnaK	7	7
NGO_10975	large pilS cassette protein	6	6
NGO_11155	large pilS cassette	8	6
NGO_1286	translation initiation factor IF-2	6	6
NGO_18311	50S ribosomal protein L16	8	6
sucC	succinyl-CoA synthetase subunit beta	5	5
NGO_0926	glutathione peroxidase	5	5
NGO_0404	restriction endonuclease%2C M subunit	4	4
NGO_07995	hypothetical protein	4	4
NGO_1046	protein disaggregation chaperone	4	4
NGO_10980	hypothetical protein	4	4
NGO_10995	fimbrial protein	4	4
NGO_11165	hypothetical protein	4	4
rpmG	50S ribosomal protein L33	4	4
NGO_1812	membrane protein	4	4
tuf	elongation factor Tu	4	4
NGO_1850	DNA-directed RNA polymerase subunit beta'	4	4
tuf	elongation factor Tu	5	4
NGO_0098	pilus assembly protein PilM	3	3
NGO_0186	butanediol dehydrogenase	3	3
NGO_0297	50S ribosomal protein L35	3	3
lldD	lactate dehydrogenase	3	3
NGO_0775	peptidase	3	3
NGO_10985	hypothetical protein	3	3
NGO_11105	pilS cassette	3	3
NGO_11115	pilin	3	3
NGO_11125	hypothetical protein	3	3
NGO_11140	hypothetical protein	3	3
NGO_11150	hypothetical protein	5	3
NGO_1358	hypothetical protein	3	3
NGO_1825	50S ribosomal protein L6	3	3
NGO_1838	50S ribosomal protein L3	4	3
NGO_1890	sodium:glutamate symporter	3	3
NGO_1908	twitching motility protein PilT	3	3
NGO_1918	UDP-N-acetylglucosamine 1-carboxyvinyltransferase	3	3
NGO_2059	trifunctional thioredoxin/methionine sulfoxide reductase A/B protein	3	3
NGO_2150	ATP synthase F0F1 subunit beta	3	3
NGO_0001	chromosomal replication initiation protein	2	2
NGO_0002	DNA polymerase III subunit beta	2	2
NGO_0058	homoprotocatechuate degradative operon repressor	2	2
NGO_0070	Opacity protein opA54	2	2
NGO_0094	pilus assembly protein PilQ	2	2
NGO_0200	phosphoenolpyruvate synthase	2	2
NGO_0372	amino acid ABC transporter substrate-binding protein	2	2
NGO_0373	cysteine ABC transporter permease	2	2
NGO_04530	hypothetical protein	2	2
NGO_0592	trigger factor	2	2
NGO_0603	integration host factor subunit beta	2	2
NGO_0614	ribonucleotide-diphosphate reductase subunit alpha	2	2
NGO_0812	hypothetical protein	2	2
NGO_0915	dihydrolipoamide dehydrogenase	2	2
NGO_0928	5-methyltetrahydropteroyltriglutamate--homocysteine methyltransferase	2	2
NGO_1049	phosphoribosylglycinamide formyltransferase	2	2
NGO_11100	Opacity protein opA54	2	2

NGO_1117	hypothetical protein	2	2
NGO_1233	ketol-acid reductoisomerase	2	2
NGO_1253	polyamine ABC transporter substrate-binding protein	2	2
NGO_1294	hypothetical protein	2	2
NGO_1308	guanosine-3'%2C5'-bis(diphosphate) 3'-pyrophosphohydrolase	2	2
NGO_1332	osmoprotectant transport activator ProQ	2	2
NGO_1344	AsmA family protein	2	2
NGO_1365	multidrug transporter	2	2
NGO_1426	iron-sulfur cluster insertion protein ErpA 2	2	2
NGO_1449	lactate permease	2	2
NGO_1457	hypothetical protein	2	2
NGO_1513	Opacity protein opA54	2	2
NGO_1528	cell division protein FtsZ	2	2
NGO_1628	hypothetical protein	2	2
NGO_1648	transposase	2	2
NGO_1767	catalase	2	2
NGO_1818	DNA-directed RNA polymerase subunit alpha	2	2
NGO_1823	50S ribosomal protein L15	2	2
NGO_1824	30S ribosomal protein S5	2	2
NGO_1832	30S ribosomal protein S3	4	2
NGO_1833	50S ribosomal protein L22	3	2
NGO_1834	30S ribosomal protein S19	3	2
fusA	elongation factor G	4	2
NGO_1855	50S ribosomal protein L11	2	2
NGO_1955	hypothetical protein	2	2
NGO_2094	molecular chaperone GroES	2	2
NGO_2119	transporter	2	2
NGO_00350	Opacity protein opA54	1	1
NGO_0044	acetyl-CoA carboxylase	1	1
carB	carbamoyl phosphate synthase large subunit	2	1
NGO_0062	formate--tetrahydrofolate ligase	1	1
NGO_0069	isoleucine--tRNA ligase	1	1
NGO_00790	membrane protein	1	1
NGO_00795	hypothetical protein	1	1
NGO_0096	pilus assembly protein PilO	1	1
NGO_0155	hypothetical protein	1	1
NGO_0172	tRNA (guanine-N1)-methyltransferase	4	1
NGO_0189	preprotein translocase subunit SecD	1	1
NGO_0201	transposase	1	1
NGO_0217	iron ABC transporter substrate-binding protein	1	1
NGO_0253	hypothetical protein	1	1
NGO_0335	polynucleotide phosphorylase/polyadenylase	1	1
NGO_0340	cysteine synthase	1	1
NGO_0349	DNAase	1	1
NGO_0371	peroxidase	1	1
NGO_03975	hypothetical protein	1	1
NGO_0407	restriction endonuclease	1	1
NGO_0420	hypothetical protein	1	1
NGO_04980	Opacity protein opA54	1	1
NGO_0523	membrane protein	1	1
NGO_0528	transposase	1	1
NGO_0530	long-chain fatty acid--CoA ligase	1	1
NGO_0562	dihydrolipoamide dehydrogenase	1	1
NGO_05620	Opacity protein opA54	1	1
NGO_0574	carbonate dehydratase	1	1
eno	enolase	1	1
NGO_0628	transposase	1	1
NGO_0629	DNA gyrase subunit A	1	1
gatB	glutamyl-tRNA amidotransferase	1	1
NGO_06725	Opacity protein opA54	1	1
NGO_0675	restriction endonuclease NlaIV	1	1
NGO_0690	lipoprotein	1	1

NGO_06995	hypothetical protein	1	1
NGO_0714	phosphogluconate dehydratase	1	1
NGO_0715	glucose-6-phosphate dehydrogenase	1	1
NGO_0719	glucose-6-phosphate isomerase	1	1
NGO_0747	tetratricopeptide repeat family protein	1	1
NGO_0766	peptidylprolyl isomerase	1	1
NGO_07675	hypothetical protein	1	1
NGO_07725	Opacity protein opA54	1	1
NGO_0777	transcriptional regulator HU subunit alpha	1	1
NGO_08230	Opacity protein opA54	1	1
NGO_0863	hypothetical protein	1	1
NGO_0872	anthranilate synthase component I	1	1
NGO_0884	hypothetical protein	1	1
NGO_0921	fumarate reductase	1	1
NGO_0959	universal stress protein	1	1
NGO_0977	acetate kinase	1	1
NGO_1006	hypothetical protein	1	1
NGO_1043	lipoprotein	1	1
NGO_1082	isocitrate dehydrogenase	1	1
NGO_1152	ABC transporter substrate-binding protein	1	1
NGO_1218	glutaminyl-tRNA synthetase	1	1
NGO_1225	peptidylprolyl isomerase	1	1
NGO_1237	ahpC/TSA family protein	1	1
NGO_1240	histidinol dehydrogenase	1	1
NGO_1256	transposase	1	1
NGO_1291	hypothetical protein	1	1
NGO_1297	hypothetical protein	1	1
NGO_1397	copper-binding protein	1	1
NGO_1406	glycine cleavage system protein T	1	1
NGO_1412	transposase	1	1
NGO_1474	LysR family transcriptional regulator	1	1
NGO_1494	polyamine ABC transporter substrate-binding protein	1	1
NGO_1507	4'-phosphopantetheinyl transferase	1	1
NGO_1508	pyridoxine 5'-phosphate synthase	1	1
NGO_1529	cell division protein FtsA	1	1
NGO_1549	cell division protein FtsN	1	1
NGO_1610	transaldolase	1	1
NGO_1656	hypothetical protein	1	1
NGO_1665	branched-chain amino acid aminotransferase	1	1
NGO_1779	Fur family transcriptional regulator	1	1
NGO_1813	LysR family transcriptional regulator	1	1
NGO_1814	cell division topological specificity factor MinE	1	1
NGO_1821	30S ribosomal protein S13	1	1
secY	preprotein translocase subunit SecY	1	1
NGO_18241	50S ribosomal protein L18	1	1
rplX	50S ribosomal protein L24	2	1
NGO_1837	50S ribosomal protein L4	3	1
rpsJ	30S ribosomal protein S10	1	1
NGO_1844	30S ribosomal protein S7	3	1
NGO_1845	30S ribosomal protein S12	1	1
NGO_1854	50S ribosomal protein L1	1	1
NGO_1870	methionyl-tRNA formyltransferase	1	1
NGO_1919	phosphoglycerate kinase	1	1
NGO_1933	amidase	1	1
NGO_1948	membrane protein	1	1
NGO_1975	30S ribosomal protein S2	1	1
NGO_2014	ABC transporter substrate-binding protein	1	1
NGO_2025	30S ribosomal protein S9	1	1
NGO_2030	cytochrome B	1	1
NGO_2032	recombinase RmuC	1	1
NGO_2086	surface exposed protein	1	1
NGO_2087	transposase	1	1

NGO_2148	ATP F0F1 synthase subunit alpha	1	1
NGO_0010	hypothetical protein	1	0
NGO_0011	DNA-binding protein	1	0
NGO_00195	hypothetical protein	1	0
NGO_00200	transposase	1	0
NGO_0049	hypothetical protein	1	0
NGO_0171	50S ribosomal protein L19	2	0
NGO_0173	ribosome maturation factor RimM	1	0
NGO_0182	preprotein translocase subunit TatB	1	0
NGO_0183	preprotein translocase subunit TatA	1	0
NGO_0313	PTS sugar transporter subunit IIA	1	0
NGO_0314	serine kinase	1	0
NGO_0636	cysteine desulfurase	1	0
NGO_0637	transcriptional regulator	1	0
NGO_0673	transposase	1	0
NGO_0674	3-isopropylmalate dehydrogenase	1	0
NGO_0916	dihydrolipoamide succinyltransferase	1	0
sucA	2-oxoglutarate dehydrogenase	1	0
NGO_1157	transposase	1	0
NGO_1158	hypothetical protein	1	0
NGO_1210	hypothetical protein	1	0
NGO_1211	transposase	1	0
NGO_1317	transposase	1	0
NGO_1318	heme transporter CcmA	1	0
NGO_1827	50S ribosomal protein L5	1	0
NGO_1830	30S ribosomal protein S17	1	0
NGO_1831	50S ribosomal protein L29	2	0
NGO_1835	50S ribosomal protein L2	1	0
rplW	50S ribosomal protein L23	1	0
NGO_18926	noncoding RNA	2	0
NGO_18931	noncoding RNA	3	0
NGO_18936	noncoding RNA	2	0
NGO_19051	noncoding RNA	1	0

Supplementary Table 3. Patient #6 MG alignment

MG gene	MG protein	SLIMM_all	SLIMM_unique
p110	P110 protein	15	14
rnpB	noncoding RNA	9	9
mgpA	MgPa adhesin	9	8
MG_281	conserved hypothetical protein	4	4
ssrA	noncoding RNA	2	2
MG_075	116 kDa surface antigen	2	2
lon	ATP-dependent protease La	2	2
nox	NADH oxidase	2	2
dnaK	chaperone protein DnaK	2	2
rpoC	DNA-directed RNA polymerase%2C beta' subunit	2	2
MG_342	NADPH-dependent FMN reductase domain protein	2	2
rpoA	DNA-directed RNA polymerase%2C alpha subunit	1	1
pdhC	dihydrolipoamide acetyltransferase	1	1
pdhA	pyruvate dehydrogenase component E1%2C alpha subunit	1	1
atpD	ATP synthase F1%2C beta subunit	1	1
tuf	translation elongation factor Tu	1	1
MG_488	noncoding RNA	1	1
MG_499	noncoding RNA	1	1
rpsS	ribosomal protein S19	1	0
rplV	ribosomal protein L22	1	0

Supplementary Table 4. Patient #8 MG alignment

MG gene	MG protein	SLIMM_all	SLIMM_unique
atpA	ATP synthase F1%2C alpha subunit	5	5
rnpB	noncoding RNA	4	4
hmw1	HMW1 cytadherence accessory protein	4	4
MG_214	segregation and condensation protein B	3	3
tuf	translation elongation factor Tu	3	3
MG_032	conserved hypothetical protein	2	2
p32	P32 adhesin	2	2
polC	DNA polymerase III%2C alpha subunit%2C Gram-positive ty	1	1
rpsL	ribosomal protein S12	2	1
rplV	ribosomal protein L22	1	1
rpmA	ribosomal protein L27	1	1
MG_472	noncoding RNA	1	1
MG_514	noncoding RNA	1	1
rpsG	ribosomal protein S7	1	0
hmw2	HMW2 cytadherence accessory protein	1	0
MG_491	conserved hypothetical protein	1	0
MG_510	noncoding RNA	1	0
MG_511	noncoding RNA	1	0
MG_513	noncoding RNA	1	0

Supplemental Table 5. Top 500 most variable human transcripts identifed after Multiple Scaling Analysis

MD	gname	DC-001	DC-002	DC-003	DC-005
ENSG00000000003.14	TSPAN6	6.241362217	8.281497425	8.091869971	6.97666845
ENSG00000001461.16	NIPAL3	7.566264888	9.473348861	9.494466909	7.059833335
ENSG00000003989.16	SLC7A2	7.374589992	8.814853045	8.060390887	6.075524724
ENSG00000006432.15	MAP3K9	9.175304932	11.83308393	11.83673638	9.124123197
ENSG00000006555.10	TTC22	7.835218882	9.728532918	10.49739541	8.162786714
ENSG00000006576.16	PHTF2	6.115454418	8.782988568	8.433739306	6.100072886
ENSG00000006831.9	ADIPOR2	9.518933378	12.33690026	11.64241059	9.741694358
ENSG00000007866.18	TEAD3	5.600755397	7.178704668	7.463398683	4.895132899
ENSG00000008086.11	CDKL5	8.737897606	11.4476475	10.94656671	8.933223004
ENSG00000008516.16	MMP25	9.590752669	7.559289767	7.560097698	8.921279206
ENSG00000010017.12	RANBP9	9.121991108	11.55059834	10.79773889	9.259053126
ENSG00000011638.10	TMEM159	7.932536075	10.52436414	10.13115058	7.612154774
ENSG00000018408.14	WWTR1	6.616740496	10.05210131	9.819254989	6.898379493
ENSG00000029153.14	ARNTL2	8.372468482	11.00641488	10.73902195	8.563147903
ENSG00000035862.12	TIMP2	9.560115183	11.73514459	11.41517816	9.612212868
ENSG00000048405.9	ZNF800	6.089342997	8.556903858	8.386558694	6.568761354
ENSG00000048544.5	MRPS10	6.226002304	9.602511188	8.56991876	6.310470227
ENSG00000049283.17	EPN3	5.667799386	7.937358396	8.32855488	5.054382413
ENSG00000050393.11	MCUR1	5.782537615	8.719061376	7.975746905	5.520448008
ENSG00000052344.15	PRSS8	8.227054983	10.81394048	10.17905769	8.248415992
ENSG00000058085.14	LAMC2	8.614119073	8.03726566	6.979036232	7.248183789
ENSG00000063601.16	MTMR1	7.983531717	9.912732155	9.585167271	7.687344154
ENSG00000064042.17	LIMCH1	5.166126195	6.072287149	6.158829629	5.097732841
ENSG00000064115.10	TM7SF3	6.932431793	8.831562969	7.720358161	6.116199127
ENSG00000065054.13	SLC9A3R2	6.314298229	8.17852581	8.008565093	5.52812359
ENSG00000065923.9	SLC9A7	7.006603923	8.899252689	9.287725004	6.795710425
ENSG00000068024.16	HDAC4	8.415036291	6.605415122	7.18168418	8.793695998
ENSG00000069011.15	PITX1	8.077992298	10.26509449	10.80783377	8.430024722
ENSG00000070159.13	PTPN3	8.302683589	10.76214085	10.62851451	8.393417354
ENSG00000071051.13	NCK2	7.069929318	8.949949196	9.54744388	7.014041333
ENSG00000071205.11	ARHGAP10	6.520054845	9.365969212	9.787780606	7.141391569
ENSG00000071242.11	RPS6KA2	7.065326868	7.519721523	8.793595589	5.88800665
ENSG00000072201.13	LNX1	7.194677104	9.798787514	9.260487987	7.417748119
ENSG00000072210.18	ALDH3A2	6.606664985	8.595787703	8.95181135	6.52461698
ENSG00000072415.8	MPP5	6.656808145	9.429276515	8.961493164	6.430250117
ENSG00000072840.12	EVC	5.63149417	6.458203554	7.710336823	5.190813526
ENSG00000073060.15	SCARB1	5.527907997	6.759178003	7.337613947	4.596654334
ENSG00000073578.16	SDHA	7.362064777	9.176814069	8.781073549	6.981463453
ENSG00000074416.13	MGLL	7.783302052	10.33060589	9.28550068	7.198194706
ENSG00000074855.10	ANO8	5.248571003	6.573144613	6.899953167	4.322084299
ENSG00000075391.16	RASAL2	8.102086613	11.0891243	10.88635543	8.40840372
ENSG00000075884.12	ARHGAP15	10.13034937	7.811689693	8.349190845	9.760977766
ENSG00000076067.11	RBMS2	8.081118212	10.91936702	11.00254303	7.98500784
ENSG00000077420.15	APBB1IP	9.819745112	7.684904635	8.384316526	9.654798325
ENSG00000081026.18	MAGI3	7.220967211	10.01651556	9.780440743	7.195098497
ENSG00000081277.12	PKP1	5.635660365	9.573203139	8.543295981	5.384699763
ENSG00000081923.11	ATP8B1	8.649084682	10.68981523	10.82888362	8.394430867
ENSG00000082397.17	EPB41L3	6.877373498	8.501908185	8.685351896	9.762436055
ENSG00000085511.19	MAP3K4	7.230014367	9.156461681	9.866211402	7.21247131
ENSG00000088387.18	DOCK9	9.594788605	11.70856256	11.40536212	9.580597811
ENSG00000088726.15	TMEM40	6.594589419	10.29398153	9.810118963	7.100918551
ENSG00000088808.16	PPP1R13B	6.223445361	7.988187438	8.744500279	5.500426404
ENSG00000089220.4	PEBP1	7.327977099	10.12134424	9.225496182	7.078471694
ENSG00000089876.11	DHX32	6.876358317	9.412571978	8.833984206	6.895472512
ENSG00000090013.9	BLVRB	5.667185616	8.259503202	7.723129901	5.560797791
ENSG00000091073.19	DTX2	7.339090096	9.200280687	9.770411435	7.388426415
ENSG00000091831.21	ESR1	7.589689491	9.896419633	9.148944582	6.897313737
ENSG00000092931.11	MFSD11	6.064118555	8.995420762	7.105489205	6.239441882
ENSG00000094755.16	GABRP	8.227772931	8.401222068	10.25963012	8.858770193

ENSG00000095637.21	SORBS1	6.000870711	6.70665637	7.6867703	4.894844204
ENSG00000099139.13	PCSK5	7.158216365	9.207964952	9.071133466	6.82927415
ENSG00000099194.5	SCD	7.980752917	10.04442254	9.765241544	8.268338618
ENSG00000099204.18	ABLIM1	11.31162089	13.85585081	13.53803245	11.39332807
ENSG00000099219.13	ERMP1	7.433874402	10.29208851	9.074635397	7.14185816
ENSG00000100014.19	SPECC1L	5.797170899	7.724910701	8.539361318	6.014297607
ENSG00000100325.14	ASCC2	8.211104971	10.14504869	10.80862604	8.771395288
ENSG00000100462.15	PRMT5	7.652135723	9.957596293	9.126688324	6.976382136
ENSG00000100997.18	ABHD12	5.868968384	7.988790723	8.304307246	5.979378904
ENSG00000101187.15	SLCO4A1	7.787661935	10.0811263	9.748390506	8.051110413
ENSG00000101193.7	GID8	7.09821489	9.225082708	8.948586108	7.049826972
ENSG00000101213.6	PTK6	6.500379947	9.301546401	9.181534547	6.123683119
ENSG00000101444.12	AHCY	6.318094564	7.942375994	9.164513822	6.581614398
ENSG00000101447.13	FAM83D	5.995002083	8.917848305	9.146080154	7.199494774
ENSG00000101782.14	RIOK3	9.578977386	12.28589192	10.90876297	9.738175477
ENSG00000101916.11	TLR8	9.646870074	7.400908614	7.58826264	9.618564888
ENSG00000101928.12	MOSPD1	5.18003901	8.076071076	7.183781992	4.87904441
ENSG00000102024.17	PLS3	8.061492813	11.17903686	9.771000492	7.873061486
ENSG00000102096.9	PIM2	9.59241541	7.704691785	7.701847813	9.418838752
ENSG00000102172.15	SMS	7.882412033	10.48203382	9.347541296	7.993659273
ENSG00000102471.13	NDFIP2	5.609630106	8.756572502	7.8057407	5.767127568
ENSG00000102837.6	OLFM4	9.879687722	9.442544698	9.506221368	9.477717536
ENSG00000103335.20	PIEZO1	7.286541695	9.912174201	9.488982308	7.014170839
ENSG00000103415.11	HMOX2	6.615063303	9.101763537	8.316902342	6.189692694
ENSG00000104320.13	NBN	9.716661092	8.578895201	8.995026567	10.87269161
ENSG00000104341.16	LAPTM4B	6.376658848	7.111388526	5.239210206	5.526365603
ENSG00000104361.9	NIPAL2	5.666391908	8.275417646	7.580559974	5.415540023
ENSG00000104368.17	PLAT	10.52168082	9.384358882	9.498000883	10.03279061
ENSG00000104413.15	ESRP1	7.398160905	9.943565463	9.129097729	6.789872541
ENSG00000104611.11	SH2D4A	6.888492345	9.921576999	7.300762373	6.608342526
ENSG00000104881.15	PPP1R13L	5.645339887	8.742614537	8.748828964	5.332589741
ENSG00000104969.9	SGTA	6.420463873	8.741146032	7.40754115	6.578131734
ENSG00000105131.7	EPHX3	5.716054499	8.958900706	9.200550087	6.40497107
ENSG00000105427.9	CNFN	6.616440778	9.707183878	9.761375495	7.510704987
ENSG00000106299.7	WASL	8.004494632	10.93197313	10.1717847	8.308758683
ENSG00000106351.12	AGFG2	5.974990683	7.430033439	9.198675784	6.06248599
ENSG00000107201.9	DDX58	8.974664532	7.251937288	7.839531453	9.883066185
ENSG00000107362.13	ABHD17B	6.581828527	10.26767745	8.819263931	6.676135772
ENSG00000107864.14	CPEB3	6.113294004	8.378195166	7.837675963	6.115346476
ENSG00000108091.10	CCDC6	7.684090208	10.31144298	9.866810312	7.590702172
ENSG00000108375.12	RNF43	5.25737198	8.206110643	7.501298681	4.78929711
ENSG00000108828.15	VAT1	7.754275356	10.39734765	10.54832208	7.993800534
ENSG00000108839.11	ALOX12	9.07462218	11.91626062	11.3140649	9.141323493
ENSG00000109189.12	USP46	5.310879906	7.369250726	7.190505883	4.74960618
ENSG00000109686.17	SH3D19	7.075408385	10.11693443	10.03323613	7.031492635
ENSG00000110429.13	FBXO3	6.92304491	9.754429626	7.718590629	6.523654971
ENSG00000110723.11	EXPH5	7.59423974	9.537867776	9.64823346	7.24507419
ENSG00000110851.11	PRDM4	7.208019505	9.76644249	9.955512103	7.508638219
ENSG00000111057.10	KRT18	8.544862826	7.393375865	7.564877833	7.539922487
ENSG00000111145.7	ELK3	6.56384015	9.618929874	9.003917247	7.10840489
ENSG00000111961.17	SASH1	10.37792141	12.80128212	12.86831377	10.64860608
ENSG00000112033.13	PPARD	7.967275219	10.83265586	10.64153735	8.57271378
ENSG00000112237.12	CCNC	6.577784326	9.292569924	8.167751856	6.421424106
ENSG00000112297.14	AIM1	11.00932586	13.53476893	12.77443569	11.01702323
ENSG00000112378.11	PERP	8.685682741	11.61193375	10.19556739	8.636767676
ENSG00000112419.14	PHACTR2	8.350648747	11.28886311	11.11231287	8.656785986
ENSG00000112699.10	GMDS	6.827516529	8.057856883	8.227594086	6.204093078
ENSG00000113645.14	WWC1	7.380113274	10.43808081	10.39034393	7.435181576
ENSG00000114019.14	AMOTL2	7.210193033	9.525857418	9.721521432	7.224679281
ENSG00000114166.7	KAT2B	8.786411901	10.88434904	10.8956635	8.807460699
ENSG00000114541.14	FRMD4B	10.41096496	8.393556585	8.462314354	10.10830476
ENSG00000114737.15	CISH	6.729805513	9.119664163	8.648289702	6.543007632

ENSG00000115009.11	CCL20	7.33258494	6.835632979	7.454614427	8.317228655
ENSG00000115607.9	IL18RAP	10.55662595	8.009679502	8.206367688	10.33335822
ENSG00000115641.18	FHL2	6.309703914	7.739169243	5.345864087	5.395737992
ENSG00000116005.11	PCYOX1	7.252185832	9.123816025	8.492960804	6.632685254
ENSG00000116016.13	EPAS1	6.909919612	8.750509343	8.897857824	6.779720134
ENSG00000116133.11	DHCR24	8.204932404	6.808509257	7.56211811	7.53504266
ENSG00000116273.5	PHF13	5.624170043	8.020497215	7.584261097	5.37434918
ENSG00000116285.12	ERRFI1	7.907019835	11.19114512	9.986956666	7.777773296
ENSG00000117054.13	ACADM	6.413880073	8.890488501	7.734957844	6.943486675
ENSG00000117091.9	CD48	9.022289345	7.294781563	7.81357643	9.626324207
ENSG00000117592.8	PRDX6	7.195136644	9.554163979	8.477392103	7.143391051
ENSG00000117595.10	IRF6	6.755106032	9.089462171	7.957639233	6.520839395
ENSG00000117620.13	SLC35A3	6.62595628	9.892660806	8.775766724	6.48386405
ENSG00000118242.15	MREG	5.664106319	8.618523173	7.772090968	6.151130853
ENSG00000118523.5	CTGF	5.681790277	6.333762336	6.910603166	5.425155051
ENSG00000118898.15	PPL	12.65160555	14.93458593	14.73191148	12.82843847
ENSG00000119508.17	NR4A3	8.804446553	7.733457949	8.406014878	9.846282544
ENSG00000119938.8	PPP1R3C	10.21951844	13.16012533	12.36962953	10.54471695
ENSG00000119950.20	MXI1	7.197928868	9.561759833	8.311320629	6.59413814
ENSG00000119979.16	FAM45A	7.719881253	10.11047539	9.586273918	7.663802945
ENSG00000119986.6	AVPI1	6.862197657	10.69354234	9.663193997	7.113001898
ENSG00000120306.9	CYSTM1	8.745287335	6.718033161	7.309575369	8.573926813
ENSG00000120756.12	PLS1	7.206201833	8.676964267	7.294775442	6.786965432
ENSG00000120992.17	LYPLA1	8.088782849	10.91594393	10.03288457	8.111548778
ENSG00000121552.3	CSTA	7.801712845	10.5445878	8.549717059	7.436446635
ENSG00000121797.9	CCRL2	9.604504782	8.260042308	9.179448731	10.4909464
ENSG00000121957.12	GPSM2	5.867078177	8.984235524	7.727035532	5.066907485
ENSG00000122042.9	UBL3	8.680795653	11.77037643	10.2807427	8.914434744
ENSG00000122547.10	EEPD1	6.499010993	8.621470518	7.866052882	5.910518877
ENSG00000122642.10	FKBP9	6.639777664	7.823061176	8.011840005	5.850101722
ENSG00000122643.18	NT5C3A	7.523790804	10.83118791	9.425055596	7.845788211
ENSG00000123595.7	RAB9A	7.324154023	9.73806487	8.260350997	6.879850711
ENSG00000123836.14	PFKFB2	8.585628186	11.53891783	10.61561522	8.588705108
ENSG00000124171.8	PARD6B	7.40924608	10.18313548	10.13462073	7.652702343
ENSG00000124882.3	EREG	7.373201	8.059616286	8.702163397	9.981284127
ENSG00000125249.6	RAP2A	6.326276853	8.640981969	7.915119912	6.408451272
ENSG00000125266.6	EFNB2	5.74855535	7.721650123	8.106800435	5.411884116
ENSG00000125534.9	PPDPF	5.886907829	9.669334852	8.90652565	5.911431949
ENSG00000125648.14	SLC25A23	5.842216381	7.009383473	8.500070656	6.098465728
ENSG00000125733.17	TRIP10	8.454557717	10.33040321	10.92272167	8.928211625
ENSG00000125735.10	TNFSF14	8.932784383	7.230123047	7.653696174	9.238790154
ENSG00000125775.14	SDCBP2	7.221347403	9.268714663	9.126391582	6.824677219
ENSG00000125780.11	TGM3	9.124635355	11.67067045	10.95445663	9.368705965
ENSG00000125868.15	DSTN	6.824129955	9.747490871	7.796092698	6.842305583
ENSG00000126368.5	NR1D1	6.633422037	10.40133367	10.0791829	6.819647525
ENSG00000126878.12	AIF1L	8.985864572	11.97766353	11.93033978	9.306170159
ENSG00000127666.9	TICAM1	6.931843522	9.642619601	9.983140015	7.461833559
ENSG00000127951.6	FGL2	9.588800343	7.372618668	7.750213095	9.550715564
ENSG00000128383.12	APOBEC3A	10.02663911	7.670967432	7.943272114	10.03026242
ENSG00000128510.10	CPA4	7.356019815	10.6901578	10.77650529	7.746021541
ENSG00000128849.10	CGNL1	7.050250129	10.74460697	10.54970264	7.259968701
ENSG00000128965.11	CHAC1	7.887131648	10.34051079	10.47071619	8.283041989
ENSG00000129451.11	KLK10	7.935604065	11.05603568	10.15447003	8.23665105
ENSG00000129521.13	EGLN3	7.574698042	10.39316576	9.533623448	6.698438231
ENSG00000130158.13	DOCK6	5.707899588	6.525733366	6.553870492	4.662605737
ENSG00000130234.10	ACE2	5.84162976	7.733624268	7.38370917	4.963717961
ENSG00000130340.15	SNX9	9.709053958	12.26555458	11.9754154	10.22564428
ENSG00000130559.18	CAMSAP1	7.21276043	8.989689178	9.535577154	7.257360479
ENSG00000130766.4	SESN2	8.25196928	10.66225109	10.53282539	8.23172267
ENSG00000130770.17	ATPIF1	5.17633959	7.309811275	5.440239073	5.014917896
ENSG00000130772.13	MED18	6.703425229	8.764341963	7.971733978	6.475625183
ENSG00000130787.13	HIP1R	6.361564008	7.888317118	7.855964381	5.662835815

ENSG00000130818.11	ZNF426	9.602615114	11.98282005	11.82487397	9.947086012
ENSG00000130821.15	SLC6A8	6.691902504	8.697253091	8.624216504	6.05872145
ENSG00000131037.14	EPS8L1	9.570908258	11.77163174	11.841481	9.66239056
ENSG00000132003.9	ZSWIM4	6.025854697	8.196272613	9.017484513	6.830492668
ENSG00000132388.12	UBE2G1	8.429490504	11.39070401	10.57322324	8.689754987
ENSG00000132698.14	RAB25	6.068243825	6.876321875	7.767193723	5.039751813
ENSG00000133030.19	MPRIP	9.006848656	11.03012976	10.96185049	8.783595957
ENSG00000133121.20	STARD13	5.247569724	7.635765777	7.661716058	5.313752652
ENSG00000133401.15	PDZD2	7.679295106	9.136638467	9.994533933	7.456601276
ENSG00000133985.2	TTC9	9.829103368	11.75927357	12.30826539	10.14985724
ENSG00000134278.15	SPIRE1	7.374442843	10.21367203	10.47153604	7.311076121
ENSG00000134317.17	GRHL1	8.215407891	10.76582178	9.935444995	8.04904569
ENSG00000134324.11	LPIN1	9.786996144	12.13218822	11.89855331	10.03715674
ENSG00000134531.9	EMP1	13.0319533	15.65117069	15.60624501	13.37129404
ENSG00000134709.10	HOOK1	5.102339113	7.392149226	5.271386704	4.555416331
ENSG00000134909.18	ARHGAP32	8.04224845	10.30300899	9.491916561	7.769817049
ENSG00000134955.11	SLC37A2	5.599736501	8.311910315	8.976266283	6.751559142
ENSG00000135052.16	GOLM1	7.929457832	6.787042532	7.119764173	7.629098021
ENSG00000135127.11	BICDL1	6.38230725	8.073436529	8.681005297	6.201453849
ENSG00000135241.16	PNPLA8	9.43704766	7.611182938	7.892842073	9.868171607
ENSG00000135245.9	HILPDA	8.803199745	12.06085527	11.32624702	9.28781495
ENSG00000135476.11	ESPL1	5.290709666	7.930915228	8.332887124	4.913601182
ENSG00000135540.11	NHSL1	6.497585161	8.492151171	9.343956486	6.398179135
ENSG00000135631.16	RAB11FIP5	6.00127127	8.408448849	8.328659862	5.561774945
ENSG00000136237.18	RAPGEF5	7.353190598	8.609214862	9.048056252	6.777667446
ENSG00000136379.11	ABHD17C	7.082421595	9.309313327	9.276308847	7.629559616
ENSG00000136542.8	GALNT5	7.457298795	6.885430896	6.122314425	6.874576296
ENSG00000136560.13	TANK	9.606861902	8.050060418	8.959328237	10.16376428
ENSG00000136636.12	KCTD3	6.357255972	8.730024187	7.837411762	6.01181814
ENSG00000136694.8	IL36A	8.858963448	9.935508365	10.82752488	9.658805464
ENSG00000136826.14	KLF4	7.052665358	9.211980077	8.740922671	6.63374212
ENSG00000137269.14	LRRC1	5.914410787	8.056166147	7.561894789	5.096012398
ENSG00000137309.19	HMGA1	8.910323465	11.23120469	11.35548229	9.063845165
ENSG00000137801.10	THBS1	9.918496608	9.705648403	9.278664376	12.28242688
ENSG00000137831.14	UACA	5.61066343	8.03929755	6.854177221	5.484289051
ENSG00000137878.16	GCOM1	6.668594192	10.18753531	10.27433427	7.43666678
ENSG00000138463.8	DIRC2	7.809605801	10.24628872	9.024946703	7.557301794
ENSG00000138593.8	SECISBP2L	9.298657272	11.64968641	11.40955346	9.453255223
ENSG00000138594.13	TMOD3	10.65664028	13.12619725	12.22420235	10.83778898
ENSG00000138623.9	SEMA7A	8.386111774	9.005797716	9.376538195	7.238960754
ENSG00000138670.17	RASGEF1B	9.012798595	11.77510969	10.72095508	9.477317227
ENSG00000138764.13	CCNG2	9.169423376	11.67997629	11.40921331	9.711838611
ENSG00000138771.14	SHROOM3	6.80357826	7.016516985	8.151529977	6.58858774
ENSG00000139116.18	KIF21A	5.753140402	8.428269842	7.322864233	5.13363812
ENSG00000139433.9	GLTP	9.058055295	11.17083431	11.39338384	9.370791843
ENSG00000139517.7	LNX2	6.405642417	8.397111831	9.11644376	5.832691522
ENSG00000140263.13	SORD	5.870047747	9.387317536	7.779032845	5.554136608
ENSG00000141098.12	GFOD2	7.642052707	11.1817203	10.47360469	7.66227776
ENSG00000141232.4	TOB1	8.118780892	11.11428506	10.27214892	8.157692269
ENSG00000141441.15	GAREM1	5.540111552	7.035679322	7.195209989	5.074481995
ENSG00000141447.16	OSBPL1A	5.875590918	9.677646527	8.490457774	5.813258073
ENSG00000141682.11	PMAIP1	6.841972127	9.79271078	8.166184302	7.762167662
ENSG00000141738.13	GRB7	5.41394997	7.720145622	7.290916622	5.03830689
ENSG00000141985.9	SH3GL1	7.208956245	9.328518545	9.733083151	7.358362391
ENSG00000142623.9	PADI1	7.94479625	10.21852454	10.65214099	8.320054363
ENSG00000142949.16	PTPRF	7.832291092	6.415700185	6.061531284	6.429282799
ENSG00000143079.14	CTTNBP2NL	7.711102572	10.29626866	9.980524035	7.68161098
ENSG00000143367.15	TUFT1	7.806943818	10.53493911	10.35336995	7.676016781
ENSG00000143369.14	ECM1	10.78988834	13.09233054	12.51764166	11.20436374
ENSG00000143382.14	ADAMTSL4	7.426746426	9.107028895	9.141372053	7.498368054
ENSG00000143412.9	ANXA9	5.164951187	8.438539635	6.793201134	4.527833931
ENSG00000143536.7	CRNN	11.69052933	14.04550299	13.45009894	11.84033104

ENSG00000143850.13	PLEKHA6	6.129632565	9.103959171	9.024368107	5.581285096
ENSG00000143878.9	RHOB	7.893834244	9.856254158	9.945444895	7.604931555
ENSG00000143882.10	ATP6V1C2	6.206609186	10.1086026	9.479948844	6.429519484
ENSG00000143995.19	MEIS1	7.754446858	6.264752329	5.53143396	6.498277401
ENSG00000144036.15	EXOC6B	6.55313283	8.319079287	7.805559343	6.033536266
ENSG00000145390.11	USP53	8.38056748	11.69136169	10.76739345	8.319051983
ENSG00000145780.7	FEM1C	7.808222723	11.220584	9.44077493	8.246754235
ENSG00000145879.10	SPINK7	6.510043462	10.2610682	8.933695684	6.698335518
ENSG00000146072.6	TNFRSF21	8.316980613	10.70385696	10.18703758	8.443284367
ENSG00000146094.13	DOK3	9.15863386	6.698792695	7.561594219	8.426531715
ENSG00000147123.10	NDUFB11	5.592007062	8.111267301	8.011270992	5.297288433
ENSG00000147394.18	ZNF185	9.988876646	12.4740008	11.93455441	10.07788479
ENSG00000147676.13	MAL2	7.101642485	8.89615667	7.680950329	5.72248371
ENSG00000147689.16	FAM83A	8.639563186	10.7106623	11.26563086	9.060666141
ENSG00000147813.15	NAPRT	6.234021469	7.312146738	7.949343793	5.725362272
ENSG00000147883.10	CDKN2B	7.127889242	8.86326935	9.49069948	7.433734667
ENSG00000147894.14	C9orf72	10.19838576	8.397677242	9.187373887	10.85847051
ENSG00000148344.10	PTGES	5.633316647	6.044391453	5.159087581	5.15128147
ENSG00000148429.14	USP6NL	8.63310152	11.16405981	11.04622602	8.948065321
ENSG00000148498.15	PARD3	7.75363728	9.515545654	9.762511801	7.179326915
ENSG00000149639.14	SOGA1	6.328534096	6.850475989	7.995758095	6.210446158
ENSG00000150977.10	RILPL2	9.203703598	7.621199661	7.928539845	9.99409046
ENSG00000151117.8	TMEM86A	5.65727356	9.424786092	9.121386397	5.523679075
ENSG00000151208.16	DLG5	7.564863581	8.528567595	7.587500147	5.954509769
ENSG00000151276.23	MAGI1	5.778812892	7.730964213	7.527941851	5.038997595
ENSG00000151693.9	ASAP2	7.635761949	10.47966777	9.763530162	7.024934654
ENSG00000151718.15	WWC2	6.096180431	8.805189478	8.290043316	6.222360183
ENSG00000151948.11	GLT1D1	9.46117582	7.251961739	8.190297227	9.270997617
ENSG00000152104.11	PTPN14	6.138581315	9.644811354	9.18187621	5.215201688
ENSG00000152242.10	C18orf25	9.699204769	12.53388818	11.74920431	9.896307914
ENSG00000152409.8	JMY	8.207630623	10.75160794	8.806145099	8.13408193
ENSG00000152944.8	MED21	5.568105004	9.109953767	7.048468498	5.368951979
ENSG00000153048.10	CARHSP1	6.787336327	8.857280578	8.618754269	6.351266665
ENSG00000153721.18	CNKSR3	5.965260319	9.682830761	9.231668163	5.510555217
ENSG00000154274.14	C4orf19	7.60801463	9.66326234	8.623732897	7.068367169
ENSG00000154319.14	FAM167A	5.70199844	8.032416279	8.831274998	5.737370389
ENSG00000155252.13	PI4K2A	6.712637448	9.612399496	10.20379653	7.30341351
ENSG00000156026.14	MCU	8.245669728	10.89926172	10.01813969	7.983663449
ENSG00000156110.13	ADK	5.570956455	8.036532013	7.178308508	5.158939439
ENSG00000156413.13	FUT6	6.837341893	9.58227022	9.697888824	6.78113984
ENSG00000157224.15	CLDN12	6.409559255	9.329935683	6.007761404	5.444771415
ENSG00000157617.16	C2CD2	6.012757015	7.553140405	9.222013523	5.619566475
ENSG00000157625.15	TAB3	8.274365686	10.54604002	10.22519488	8.506749639
ENSG00000158246.7	FAM46B	5.472623385	9.211306707	8.673642028	4.888230932
ENSG00000158825.5	CDA	6.430934042	8.784963996	6.908336181	5.21469572
ENSG00000159166.13	LAD1	7.096705371	8.845757761	8.941399205	6.365912939
ENSG00000159363.17	ATP13A2	5.340761671	7.95858869	7.907338076	5.254458706
ENSG00000160213.5	CSTB	10.63457847	13.32796231	12.12773129	10.73118504
ENSG00000160352.15	ZNF714	5.780355041	8.849646571	7.634407212	6.030471837
ENSG00000160439.15	RDH13	6.494897399	9.729106747	9.735111753	6.459076076
ENSG00000161091.12	MFSD12	6.504693061	8.686627861	7.029672736	6.125007026
ENSG00000161249.20	DMKN	6.556793814	9.750312337	8.598689723	6.824145274
ENSG00000162032.15	SPSB3	5.934053276	8.332136514	8.395474211	5.776326277
ENSG00000162069.14	BICDL2	5.356762157	7.455716435	7.399511779	4.733241929
ENSG00000162104.9	ADCY9	7.466031115	10.15616573	10.83575506	7.161140501
ENSG00000162542.13	TMCO4	6.812792741	8.537882973	8.040763532	5.596259673
ENSG00000162852.13	CNST	8.402054551	11.22002659	11.04503582	8.642307008
ENSG00000162981.13	FAM84A	6.695942433	9.407836779	9.363012976	6.932767688
ENSG00000163202.4	LCE3D	10.66170784	13.2166533	12.08627642	10.73079222
ENSG00000163209.14	SPRR3	13.44500598	16.17384278	15.17458235	13.73341946
ENSG00000163216.6	SPRR2D	11.91408625	14.46414357	13.969992	12.27086803
ENSG00000163347.5	CLDN1	6.657258773	7.499147983	5.753843494	5.507270668

ENSG00000163624.5	CDS1	7.200022447	9.754471918	9.659135164	7.330718985
ENSG00000164096.12	C4orf3	7.978169336	10.62168485	9.367987307	8.479544673
ENSG00000164120.13	HPGD	8.165345382	11.09355532	9.488910722	8.347352979
ENSG00000164142.15	FAM160A1	6.950590611	7.581520224	8.816836851	6.291199593
ENSG00000164284.14	GRPEL2	8.175501646	9.985267342	10.61898781	8.867973626
ENSG00000164379.6	FOXQ1	5.177335836	8.060287146	6.836473576	4.834137064
ENSG00000164442.9	CITED2	8.848490806	12.05399113	10.80897625	8.859526412
ENSG00000165272.14	AQP3	6.376550866	6.960250592	5.690378336	5.025565323
ENSG00000165527.6	ARF6	8.487963098	10.74753266	10.43255217	8.84497785
ENSG00000165732.12	DDX21	10.03877334	8.381545002	8.761490085	10.72953396
ENSG00000165799.4	RNASE7	10.65007333	13.15547905	12.94993432	10.95432262
ENSG00000165997.4	ARL5B	9.528052148	8.355780071	8.661287705	10.68741454
ENSG00000166016.5	ABTB2	6.638534919	9.416664027	9.958315153	7.443310658
ENSG00000166165.12	CKB	5.166740576	6.762931369	5.063808818	3.993485875
ENSG00000166396.12	SERPINB7	7.944539399	9.986662884	7.459205931	5.412818975
ENSG00000166398.12	KIAA0355	6.961605689	9.749015491	9.649176436	7.014712288
ENSG00000166439.5	RNF169	10.27526813	12.45856918	12.12302872	10.3578885
ENSG00000166535.19	A2ML1	12.24249371	14.50793085	13.9382743	12.29605246
ENSG00000166557.12	TMED3	6.698695534	7.255379133	8.138502637	5.794534298
ENSG00000166828.2	SCNN1G	7.186368016	9.054373349	8.397119709	6.801918239
ENSG00000166833.19	NAV2	6.350929792	7.048546402	6.33420487	4.911197522
ENSG00000167106.11	FAM102A	7.34757483	8.949930902	8.744612877	6.968524428
ENSG00000167653.4	PSCA	8.861083809	11.22249699	11.09776993	9.252347639
ENSG00000167757.13	KLK11	7.014004529	9.640397124	9.039044708	6.783374258
ENSG00000167759.12	KLK13	8.991153588	12.03949324	10.99666273	9.002902183
ENSG00000167766.18	ZNF83	7.798105588	10.33989892	8.737165951	6.607339332
ENSG00000167767.13	KRT80	8.734240814	11.63966338	10.91278166	8.616379364
ENSG00000167772.11	ANGPTL4	6.006038314	8.258835677	7.393003791	6.414999856
ENSG00000167880.7	EVPL	6.744403479	9.264899979	9.209954471	6.438963043
ENSG00000168140.4	VASN	6.317799839	9.315002532	9.576229805	6.10693527
ENSG00000168398.6	BDKRB2	5.014416186	6.525911936	6.348710338	4.403496626
ENSG00000168447.10	SCNN1B	7.873569791	10.61008624	10.04369135	7.747001196
ENSG00000168575.9	SLC20A2	6.934127601	10.04623954	9.789408893	6.951736529
ENSG00000168672.3	FAM84B	7.032758928	9.480440785	8.658813914	6.498849232
ENSG00000169035.11	KLK7	6.159764424	7.028854396	6.780066455	6.312018599
ENSG00000169129.14	AFAP1L2	6.556093674	7.914896051	8.386195974	5.953628963
ENSG00000169469.8	SPRR1B	9.933118349	12.30622601	11.37653174	10.08731967
ENSG00000169509.5	CRCT1	10.82047826	13.4385399	13.02851491	10.9731331
ENSG00000169756.16	LIMS1	8.045710692	7.095565445	8.372798957	9.189153643
ENSG00000169908.11	TM4SF1	6.784032409	8.956226574	8.172545386	6.134389855
ENSG00000170185.9	USP38	8.575095897	10.89608871	10.20203783	8.696688369
ENSG00000170423.12	KRT78	10.83002088	13.21729743	13.0322819	11.09831411
ENSG00000170477.12	KRT4	10.05069138	12.71188388	12.08926737	10.05505999
ENSG00000170545.16	SMAGP	5.623907961	8.277222167	7.713721971	5.178400326
ENSG00000170786.12	SDR16C5	5.570532894	7.407220702	5.674576545	4.977595515
ENSG00000170919.15	TPT1-AS1	7.139610853	9.31194071	8.115299119	6.987320425
ENSG00000171161.12	ZNF672	6.34822175	9.319446203	8.980279446	7.066435172
ENSG00000171346.14	KRT15	7.214651362	8.588770915	8.301282954	7.18133269
ENSG00000171466.9	ZNF562	7.660579137	10.26363709	9.225487503	7.805968159
ENSG00000171476.21	HOPX	6.149438879	9.657861021	8.834066305	6.205357813
ENSG00000171552.12	BCL2L1	8.066490578	10.55145136	10.33446074	8.101992693
ENSG00000171766.15	GATM	5.509767259	8.119230564	8.976007934	6.127368172
ENSG00000171813.13	PWWP2B	5.089327293	7.193534257	7.550160492	4.570449453
ENSG00000172331.11	BPGM	8.708216439	10.94634353	10.28951619	8.801161692
ENSG00000172380.5	GNG12	8.60645494	11.89644676	10.71668017	8.594085562
ENSG00000172382.9	PRSS27	8.089771617	9.910413046	10.61516778	8.598646085
ENSG00000172478.17	C2orf54	6.922898249	10.44010175	10.34332972	7.450525943
ENSG00000172731.13	LRRC20	5.928194718	9.694254683	8.413741649	5.703747454
ENSG00000172818.9	OVOL1	5.910982216	8.615201698	8.522442312	6.284381052
ENSG00000172819.16	RARG	6.193937421	8.77960021	9.327535816	6.117402083
ENSG00000172927.7	MYEOV	5.258335791	6.876233353	8.329070174	5.3071554
ENSG00000173065.13	FAM222B	5.169754507	7.977068876	6.26812215	5.337266421

ENSG00000173210.19	ABLIM3	6.465897221	9.627786091	8.937444433	6.03328555
ENSG00000173548.8	SNX33	7.453187564	9.139470259	9.851377538	7.643711367
ENSG00000174013.7	FBXO45	4.971071917	6.83345838	6.764686086	4.908566839
ENSG00000174306.21	ZHX3	5.502372097	7.605618811	7.505197706	4.681687802
ENSG00000174738.12	NR1D2	7.754018888	10.5490287	9.892513349	7.891786937
ENSG00000174837.14	ADGRE1	9.544660004	7.489392415	8.071484191	9.973488638
ENSG00000175137.10	SH3BP5L	6.975448366	8.897393932	9.097621317	6.628345203
ENSG00000175274.18	TP53I11	6.670222088	8.78847913	9.876698867	6.543562309
ENSG00000175662.17	TOM1L2	6.751753378	8.490259857	8.646413495	6.210111378
ENSG00000175984.14	DENND2C	6.916066901	9.980582283	9.809079271	6.925812855
ENSG00000176014.12	TUBB6	6.699977617	9.926738151	9.343632883	7.246901096
ENSG00000176092.13	AIM1L	7.70236726	10.51845655	10.6645757	7.961854606
ENSG00000176105.13	YES1	6.417345429	8.338545539	8.08725404	5.847664062
ENSG00000176171.11	BNIP3	6.686030776	10.32386299	8.678964666	6.976441737
ENSG00000176834.13	VSIG10	6.457820658	9.447555649	8.673881017	5.209441729
ENSG00000176903.4	PNMA1	5.327526899	7.700329529	7.278033102	5.222057639
ENSG00000176945.16	MUC20	7.056250762	8.593515764	8.662237653	6.087710013
ENSG00000177030.16	DEAF1	5.193079647	8.589654384	7.962295574	5.315556686
ENSG00000177042.14	TMEM80	5.825275175	9.435645066	9.34528483	6.1471747
ENSG00000177106.14	EPS8L2	7.978241582	10.00164026	10.30114503	7.676644815
ENSG00000177169.9	ULK1	6.794611212	8.932076711	8.64018914	6.309683305
ENSG00000177409.11	SAMD9L	9.53109226	7.680291966	8.334718085	10.41830551
ENSG00000177427.12	MIEF2	5.359618604	8.391438035	8.54276028	5.007243367
ENSG00000178038.16	ALS2CL	6.64010736	8.267943842	7.737167067	5.677140718
ENSG00000178075.19	GRAMD1C	6.635352175	8.878710149	7.810383136	6.196824074
ENSG00000178585.14	CTNNBIP1	6.186760493	9.341154271	7.925739637	5.804600072
ENSG00000179833.4	SERTAD2	9.433087363	12.21091411	11.78706522	9.67620575
ENSG00000179913.10	B3GNT3	6.948593548	9.536722135	9.504639232	7.01844144
ENSG00000180667.10	YOD1	10.7989182	13.49410948	13.06742743	11.13751496
ENSG00000180921.6	FAM83H	6.300152647	7.636778702	7.938709611	5.308970938
ENSG00000181333.11	HEPHL1	9.962506289	12.12811435	11.30672109	9.65189911
ENSG00000181467.4	RAP2B	7.953191472	10.7027887	10.21465282	8.657757061
ENSG00000181652.18	ATG9B	6.84418021	10.01355572	10.0527457	6.850145432
ENSG00000182372.8	CLN8	6.361268146	9.576862017	9.534532292	6.453779115
ENSG00000182795.12	C1orf116	9.353391975	11.59574623	11.73201741	9.810213761
ENSG00000182872.15	RBM10	6.963744014	8.812997043	8.64638625	6.909153424
ENSG00000182957.15	SPATA13	9.439731572	7.62296979	7.149008802	9.945161668
ENSG00000183018.8	SPNS2	10.8047691	12.68426912	13.0575449	10.91507587
ENSG00000183337.16	BCOR	7.611925168	10.05344169	9.663715862	7.761791997
ENSG00000183421.11	RIPK4	6.847841146	8.919495169	9.307330621	6.644308467
ENSG00000183696.13	UPP1	9.028150501	11.61515415	10.64539053	9.31734448
ENSG00000183723.12	CMTM4	7.273387343	6.792627805	6.395941335	6.221100533
ENSG00000183778.17	B3GALT5	7.743802499	7.550734023	10.51002472	7.470378469
ENSG00000184731.5	FAM110C	5.171501298	6.677902277	6.258926954	4.598758727
ENSG00000185567.6	AHNAK2	8.713653988	11.48813694	10.77637472	8.460718134
ENSG00000186174.12	BCL9L	7.128807243	9.460150842	9.65135685	7.115636147
ENSG00000186187.11	ZNRF1	5.257252169	7.013743161	7.885861326	5.568892438
ENSG00000186300.11	ZNF555	5.817294448	8.975585144	6.151401189	6.680105894
ENSG00000186352.8	ANKRD37	6.523049959	9.379730359	8.226500112	6.688753578
ENSG00000186522.14	10-Sep	6.143841207	9.762108213	8.331666933	6.180054884
ENSG00000186806.5	VSIG10L	6.467187126	9.65856025	10.19471475	7.275442783
ENSG00000186832.8	KRT16	8.907431021	10.79280005	10.92037514	8.929634146
ENSG00000187054.15	TMPRSS11A	7.424133395	10.53866988	9.14062078	7.901786406
ENSG00000187079.15	TEAD1	8.962358886	11.60182308	11.31049537	8.999516612
ENSG00000188001.9	TPRG1	6.205934311	9.715412935	8.823818475	6.792935498
ENSG00000188042.7	ARL4C	7.704500609	9.876920202	10.57604357	8.219276468
ENSG00000188112.8	C6orf132	9.897400927	12.48228783	12.48996459	10.05075652
ENSG00000188505.4	NCCRP1	9.700009346	11.69876913	12.13017421	10.04715559
ENSG00000189001.10	SBSN	6.415943234	9.794221125	9.98674709	6.362786258
ENSG00000189060.5	H1F0	8.322963372	10.86161977	10.06041612	8.329645287
ENSG00000189334.8	S100A14	7.208507818	7.56794013	7.951377635	6.485618034
ENSG00000189337.16	KAZN	6.692611399	9.281390433	9.693835977	7.123527945

ENSG00000196268.11	ZNF493	7.652619375	7.514945706	5.863446103	8.66499298
ENSG00000196428.12	TSC22D2	8.74803329	11.88133489	10.95713338	8.96361674
ENSG00000196549.10	MME	10.26545635	8.023444542	8.284525733	9.773677636
ENSG00000196705.8	ZNF431	7.396726923	10.52102247	9.298145241	7.467711026
ENSG00000196743.8	GM2A	10.30294013	13.05290312	11.16959618	10.36240472
ENSG00000196850.5	PPTC7	7.723451741	10.02829594	9.769951177	8.062129787
ENSG00000196935.8	SRGAP1	6.248037799	8.161762419	9.524983092	6.812209053
ENSG00000196968.10	FUT11	6.294796314	8.234019715	8.124139071	7.163480955
ENSG00000197070.13	ARRDC1	5.53920871	7.539596529	7.124373363	4.892218611
ENSG00000197077.13	KIAA1671	7.460234969	8.58180793	7.79545865	6.46123977
ENSG00000197353.3	LYPD2	5.737035898	7.733404027	8.012187467	5.832575703
ENSG00000197563.9	PIGN	5.909236636	8.98027483	7.109374013	5.962167963
ENSG00000197641.11	SERPINB13	7.855416192	10.02208436	8.524389575	7.597423698
ENSG00000197948.10	FCHSD1	7.026874296	9.659346294	7.461390326	6.594172986
ENSG00000198142.4	SOWAHC	7.783689052	11.27081146	10.61162595	8.183977093
ENSG00000198478.7	SH3BGRL2	8.961059551	12.23605918	11.2593065	9.202017405
ENSG00000198574.5	SH2D1B	5.150692744	6.37792734	5.875264677	5.145836861
ENSG00000198682.12	PAPSS2	9.612569515	12.54097016	11.59685796	10.02156417
ENSG00000198722.14	UNC13B	7.476369809	8.356319133	9.491307695	6.803214652
ENSG00000198853.11	RUSC2	6.530400236	9.849665225	10.08397945	6.838397295
ENSG00000203688.4	RP11-351J23.1	5.998542837	8.474198857	8.712283645	6.217106279
ENSG00000203709.10	C1orf132	8.749322232	6.795010211	6.581880976	8.78803187
ENSG00000203785.8	SPRR2E	11.34839903	13.69235882	12.94062859	11.668673
ENSG00000204020.5	LIPN	8.855124873	7.724001041	6.581472029	8.735367129
ENSG00000204131.8	NHSL2	9.66049025	7.360175332	7.467928493	9.559563365
ENSG00000204138.12	PHACTR4	8.715811318	11.32543809	10.92096578	9.07593033
ENSG00000204178.9	TMEM57	7.038072999	9.884080362	9.174208976	7.454460578
ENSG00000204388.6	HSPA1B	8.184847344	7.267567375	7.209369162	7.776871222
ENSG00000204618.8	RNF39	7.03301226	10.19817731	9.807535216	7.01267376
ENSG00000204839.8	MROH6	5.846753349	7.892519017	8.51516294	5.556912536
ENSG00000204936.9	CD177	9.157087419	8.522247332	9.939871304	7.761096268
ENSG00000205420.10	KRT6A	11.55673922	13.65929264	13.31163099	11.70455286
ENSG00000205476.8	CCDC85C	5.957860612	7.425383981	8.559035813	5.643147998
ENSG00000205730.6	ITPRIPL2	7.354677459	9.73168611	10.22906608	7.907846761
ENSG00000210117.1	MT-TW	7.515915403	8.369782817	8.305387391	6.031106336
ENSG00000211445.11	GPX3	7.575618573	10.64168725	9.977335749	7.847494189
ENSG00000213020.8	ZNF611	6.984029623	9.540672667	7.292855102	6.527790047
ENSG00000213064.9	SFT2D2	9.709519998	11.99324046	11.2383191	9.630969914
ENSG00000213753.10	CENPBD1P1	6.188170667	8.809462448	8.262737143	6.356258061
ENSG00000213859.4	KCTD11	6.156909748	8.88908205	8.645824697	5.342164754
ENSG00000213923.10	CSNK1E	9.270792722	11.66710046	11.5279626	9.336753053
ENSG00000214814.7	FER1L6	7.267851453	8.450541469	7.811741448	5.938877024
ENSG00000214944.9	ARHGEF28	5.770643137	8.482572574	6.818369734	4.367624953
ENSG00000221926.11	TRIM16	6.26151754	8.225659253	6.977101182	5.403786924
ENSG00000233452.6	STXBP5-AS1	5.450456647	7.587070696	7.525253808	4.957425511
ENSG00000234678.1	RP11-465N4.4	5.66990516	7.162919039	6.546003454	4.740177564
ENSG00000237973.1	MTCO1P12	6.138153381	6.475538128	9.907650383	5.503461854
ENSG00000240849.10	TMEM189	5.529847622	7.42870177	7.655694092	5.847542699
ENSG00000240891.7	PLCXD2	6.197300062	9.600412976	9.338924094	6.286610957
ENSG00000241794.1	SPRR2A	12.39701686	15.04054072	14.27945057	12.68691196
ENSG00000243317.7	C7orf73	5.710016159	8.116860208	6.452226172	5.750614295
ENSG00000244094.1	SPRR2F	8.929397656	11.29076119	10.329885	9.692298564
ENSG00000249915.7	PDCD6	7.145929599	9.784037423	9.3411926	7.288765839
ENSG00000256612.7	CYP2B7P	8.117791979	10.08382917	11.01649544	7.87612908
ENSG00000261040.6	WFDC21P	6.54054651	8.768752663	7.245297878	6.79473866
ENSG00000261068.2	RP11-7K24.3	6.556404411	10.36927488	10.18146364	6.609607106
ENSG00000261272.1	MUC22	8.8614578	11.22988453	11.15181935	9.47562803
ENSG00000261324.2	RP11-174G6.5	5.660934487	6.692911105	6.774547244	5.498848079
ENSG00000261553.5	RP11-29G8.3	5.189898509	8.588818344	7.677285003	5.206146239
ENSG00000268858.2	RP4-591C20.9	5.405517222	7.126736217	8.091411281	5.84264438
ENSG00000269825.1	CTD-3099C6.9	6.390134176	10.22355564	8.734534517	5.624401599
ENSG00000275302.1	CCL4	7.740430567	7.813481332	8.457858201	9.454085514

ENSG00000275342.4	SGK223	5.190803751	7.400264096	7.612424556	4.835065958
ENSG00000275993.2	CH507-42P11.8	6.484281875	9.300247406	9.425313486	6.500247593
ENSG00000276023.4	DUSP14	8.004212399	11.30362514	10.20886752	8.081261617
ENSG00000276070.4	CCL4L2	7.753814989	7.685807196	8.67354841	9.138695905
ENSG00000276085.1	CCL3L3	8.23564607	8.066020002	9.117426533	9.589088447
ENSG00000277496.1	RP11-93B14.9	5.567930752	9.537669207	8.085387018	5.797278477
ENSG00000278771.1	Metazoa_SRP	9.780470226	8.67245115	8.582646571	11.65217543

DC-006	DC-008	DC-009	DC-010
6.941435767	7.489506892	7.9782889	8.863561985
7.378654411	7.072982761	8.339209836	7.844753452
6.667026883	6.42701433	7.775906143	7.280058789
9.236385593	9.260252779	10.36489417	9.357384111
7.935437872	8.194905282	9.271265538	9.264257141
6.32200277	6.866071028	6.75224994	6.338062183
9.575906671	9.954175464	10.44339881	9.830823037
5.904599272	5.125214278	6.268302722	6.239218195
8.720639996	9.108310968	9.778231568	9.117523192
8.969059235	10.1577794	8.781632376	8.097386552
8.930689378	9.373522165	10.1593984	9.36305866
7.803640014	8.103843365	8.823285615	8.871066479
6.352562628	6.571856114	8.111487281	6.202796429
8.669878157	8.811977559	9.241913916	9.503263134
9.582443007	9.896532756	9.920843553	9.520531639
6.360405317	6.980095812	6.971987927	6.404769304
6.112830658	6.765959586	7.53419536	7.235649955
5.57787082	5.080216593	7.076662648	5.724900772
6.141708405	5.955660507	6.464425225	6.050199467
8.495083769	8.497368038	9.11552441	9.099670739
8.356900237	7.49945554	9.266872211	9.217552966
7.411763761	8.287962326	8.027639039	7.648174751
5.31977697	4.60885968	7.201293012	7.763888108
6.742646744	5.879350002	7.866485587	6.707821093
6.533746274	5.755495348	7.538900328	6.46555107
7.338938736	6.833712928	7.532972723	6.979386649
8.223652754	8.851744043	7.125078215	7.031614156
7.991446952	7.984689611	9.426985711	9.105782984
8.563173241	8.716420895	9.532494178	9.175706923
7.046080926	6.897548875	8.205862482	7.67234955
7.138403133	7.214173091	8.581453799	7.269643286
7.233565763	5.884308557	7.358555055	6.901876055
7.06444495	7.442887701	8.802432623	8.38909406
7.097328083	5.907254762	7.700108376	6.626114733
6.678157104	6.737116937	7.710238995	7.267541447
5.652548638	4.804537225	5.753440553	4.991600625
5.953052975	4.710657525	6.097834391	4.683069676
7.319209342	6.815243496	8.201895148	7.364136724
8.097386891	7.416474445	8.111427157	7.268733756
5.175256984	4.621205255	6.148138653	5.190330078
8.456921287	8.554248557	9.396872378	8.808383172
8.992336189	10.52744817	8.833275082	8.47809954
8.396496707	8.294240848	9.244320163	8.350434227
9.166241723	10.52780454	8.363350807	8.557383654
6.949609471	7.663210614	7.972699787	7.919518954
6.656852185	5.635427448	7.909964043	5.451887954
8.664592663	8.512000576	9.3727799	8.905902948
8.84577028	8.002513984	7.350566676	8.914564792
7.38925929	7.374437304	8.16017142	7.865615849
9.623958208	9.675188757	10.75444737	10.22610823
6.624584681	7.179758701	8.617527743	7.595325441
6.09242312	5.76657802	7.584648593	6.379113732
7.458856096	7.067651293	8.399866273	7.41335546
7.272643949	7.191571173	8.18812713	8.303738979
5.929791639	5.942643201	7.572159999	6.151366099
7.222218633	7.200718888	7.808611438	6.985315151
7.377306151	7.021432443	9.352527348	7.719714362
6.409527228	6.0921814	6.884263911	6.813587726
9.020896214	8.928068849	10.28561824	10.67535061

5.833232389	5.231089705	6.9268408	5.97858323
6.722766038	6.485951386	8.178619815	6.566879444
9.019083277	7.571155673	8.46461268	7.735515311
11.29035137	11.48765505	12.09021394	11.44835917
7.613720994	7.14919617	8.646382332	8.467644462
6.05496434	6.169321364	6.982632695	6.526364178
8.104848908	8.085490708	9.426651415	8.744834011
6.867073761	7.064515352	7.951246965	6.746788441
6.840864688	5.835198878	7.63996681	6.367324423
8.000317315	8.032149993	8.187099059	8.853017345
7.268008342	7.079665182	7.967784168	7.338942432
6.051891705	6.637686018	7.661583502	6.336654761
6.656929794	5.415095914	8.572490318	7.171573711
6.03906751	5.622857164	8.562707364	7.333768679
9.601597248	10.23535773	10.18113543	10.35276707
8.820611317	10.18835729	8.282900409	8.759395674
4.865034155	5.736472222	6.886183214	6.031646879
8.235869203	8.649076954	9.111617586	9.06708137
9.066761927	10.05410611	8.348543376	9.24352968
8.046703387	8.331087765	8.241059077	9.015273488
6.03442681	6.741815996	7.482289536	7.395330764
10.10313947	9.809794353	10.29330813	12.2451852
7.782842867	7.029613133	7.623529313	7.49507029
6.44111018	6.217190301	7.787632934	6.808592613
9.984917111	11.41421996	9.064131583	9.775196354
6.803975636	5.880082124	7.746777895	7.386527695
5.558944816	4.904978142	6.90102111	6.166575851
10.58601708	10.06519169	10.85703648	12.14245024
7.448338785	7.221193263	8.915179668	8.018176495
7.006271938	6.514325582	7.833924742	7.675569775
5.591078114	5.515717937	7.128930995	5.648444121
6.422978799	5.869753327	6.700272649	6.668508649
5.858022661	5.793131238	7.183033007	6.699963
7.023603742	7.006584686	9.129042838	8.001350617
8.360875974	8.913521404	9.198274251	9.018104604
6.313554475	6.078695609	7.575334953	7.459495575
8.477190633	9.573109024	8.581484549	8.27679551
6.559976318	7.543286723	7.861889348	7.770312767
5.749407455	6.218544353	6.9054468	6.42086448
7.95586435	7.844442039	9.270695258	8.063214027
5.204769412	4.705782406	6.713199865	5.272309497
8.19500474	7.846618227	9.020149316	8.409301238
8.944540754	9.687165273	10.30872692	9.039204837
5.965894974	5.169006627	6.802568591	6.53061836
7.047457256	7.291112492	8.164487338	7.065101297
6.528506257	6.551003144	7.031511826	6.764591185
7.689958884	7.729295297	9.085569868	8.431711876
7.457696669	7.257427164	8.307477443	7.483802756
8.39873125	7.213370626	9.893980485	9.220922888
7.126075513	7.265266603	7.550673972	7.569457693
10.3432059	10.72328507	11.47562595	10.51060565
8.405327392	8.077332167	9.078882087	8.436419326
6.298737998	6.734171363	7.490288989	7.016659236
11.02702835	11.19608776	11.6744329	11.11403345
8.735883061	9.062570839	9.6555596	9.634376331
8.677757007	9.072614093	9.189946032	9.052584213
6.40006762	5.524096276	7.631161453	6.580819327
7.557312836	7.665667414	8.578899515	7.849230839
7.522124546	7.29423829	8.329891126	8.436394359
8.440198471	9.12316236	9.069947132	8.496611492
9.001882048	10.58168238	9.070093567	9.272295649
7.470828994	7.839265837	6.845839971	6.511833577

7.592173666	8.502401751	7.132737989	9.573840748
8.905641212	10.3346782	8.982959281	9.217368167
6.157045804	5.944047818	7.033028214	7.660165907
7.391726546	7.016215868	8.611560943	8.010422136
7.359521582	6.587945451	8.09920165	7.461621338
8.147350226	6.755990984	9.321131148	9.325253469
5.890840514	6.057766229	6.318382938	6.137771571
8.140953017	8.178670552	8.837721955	8.840931763
6.117042559	6.780883342	7.88867153	8.288408636
8.71371609	9.966156387	7.654527802	8.948147938
7.403050295	7.109718044	7.821249357	7.107935783
7.489691094	6.897971741	8.23178452	8.268223379
6.551231639	7.296683628	7.563059656	7.020986892
6.674778345	6.155717668	7.0150248	6.831294628
6.45912617	5.504998461	9.315961038	5.584272752
12.69732541	12.81046176	13.75160032	13.08977973
8.143047933	10.12288111	7.844729663	9.012014027
10.17031667	10.30416395	11.64155007	10.84920063
6.79751009	7.733526672	7.570053861	7.85750814
7.410992874	8.191288513	8.197728862	7.594744832
6.941434392	7.002349104	8.320970729	8.038532949
8.051607037	9.383982764	7.329257055	7.553923614
6.94400311	6.720211682	8.420414699	8.687141124
7.965486523	8.361150473	8.910731764	8.728153891
7.459030333	8.372585549	8.931005605	8.745550014
9.688268528	10.49459761	8.153486921	9.808861112
5.669292393	5.959012672	6.752606756	6.550286291
8.771407638	9.222445534	9.430370415	9.197644133
5.993863068	6.308590789	6.50406844	6.089850245
6.80576723	5.368668013	7.398260297	6.615496122
7.177823275	8.220213509	7.934759756	7.954475578
6.946792442	8.16167405	7.717020552	8.320003432
8.534747237	8.961815484	9.354252583	8.512272505
7.915412151	8.308381817	9.169964922	9.154851668
8.17486742	9.308921499	7.89716532	9.381681335
6.938041143	6.18379336	6.52477319	6.674161443
5.767521723	5.91933872	6.164531351	7.773221015
5.666254427	6.596358421	7.497056617	6.301625664
6.118472081	6.259901847	7.987231912	7.021981435
8.715859478	8.720237962	9.650004951	9.42188416
8.146505837	9.690611666	7.640640153	8.417730672
7.161372447	7.231804844	8.372109394	8.38112921
8.828997971	9.13448776	11.84673299	8.706723262
7.249005382	7.570861573	8.013323047	8.174878987
6.62929644	7.532544114	7.979452362	7.157226339
8.96909481	9.49649402	10.36025222	9.554423078
6.728674518	7.152228832	7.605369415	7.056237961
8.593233164	9.634527215	8.387546375	8.972334699
9.309350969	9.680703315	8.889626101	9.373566158
7.343209979	8.087371226	9.969967964	7.977279871
7.043178722	7.461863158	8.974973292	8.122127313
7.873858632	7.954905563	8.903684811	8.69371179
8.234329865	8.144212681	9.057762575	8.737108068
6.9512346	7.278643824	8.015357229	7.631937723
5.657108115	4.50913135	6.456811058	4.472163965
5.821319726	6.456305109	7.346281133	7.407774559
9.875066652	10.14930947	10.61318612	10.35597698
7.718217963	6.8692003	8.445108402	7.520022085
7.933782671	8.37093906	9.558404066	8.656428411
5.668845536	4.581338106	6.50263536	5.653694052
6.235973816	6.268286218	7.369964216	6.506797167
6.528211914	5.599312746	7.093129812	7.197674615

9.546562175	10.15892843	10.66744198	10.32979773
6.015386967	5.339963897	7.86007891	5.798315761
9.667478049	9.834735577	10.68470591	10.17332965
6.901002387	6.929079692	7.388190506	7.045911141
8.458548712	8.920777549	9.760552171	8.716110639
5.988846371	5.137835523	7.590076512	6.825273578
9.210421436	8.750553486	9.92635444	9.316575987
5.518671127	4.651786734	5.393660142	4.937872431
7.963877027	7.347444236	9.128694259	8.3641406
9.835704923	10.05226612	10.66588331	10.38105208
7.48247713	7.640008585	8.422931194	7.218362022
8.422571442	8.586042747	9.567605796	9.509185892
10.09547368	9.97012456	11.13785468	9.824362748
13.12300865	13.37371885	14.22450242	13.55800124
5.355074269	4.944509858	6.082395038	5.713364846
8.459934124	8.152949484	9.405567653	9.083451871
7.333234276	5.911805865	6.849234581	6.692963268
8.288035253	7.801790826	8.645538421	9.706477839
6.214593852	5.543171705	6.555968337	6.984096311
8.646637854	10.27621809	8.538138754	9.214770258
8.725685567	9.300645525	10.33771769	9.207142409
4.467779968	5.689397161	6.968923458	4.836818527
5.913170227	5.849995311	8.057923632	6.379498692
6.026122579	5.778196788	7.454413956	6.633882649
7.124565458	6.901134155	8.334425838	8.081910911
6.969124127	6.992547365	8.554671819	8.190876531
7.629204684	7.926010939	7.581404568	9.691121082
9.4578037	10.72917896	8.522462402	9.302428795
6.780543852	6.660594994	7.58141595	8.047815538
8.634008745	8.378880767	10.73291224	9.397938928
6.934006875	6.924149203	7.719873148	8.038945225
6.196321153	5.832634896	6.688995198	6.796111293
9.163109226	8.981492618	9.935090455	9.157100143
10.00578872	10.09764091	9.50029726	11.29841871
6.119993337	7.051949044	7.284968709	6.661715639
6.569988103	7.258356831	8.438988715	7.965451751
7.613284927	8.236400364	7.854023695	7.938651263
9.383563209	9.650018708	10.02754788	9.854111255
10.66282831	11.04305708	11.08621475	10.88188401
7.568011086	6.795511931	8.535237902	7.328599333
9.319030477	9.934170242	9.898827826	9.743043468
9.241960759	9.834304996	9.984591163	9.583506845
6.462423752	5.515651393	8.660955768	7.329785618
5.694986651	5.579867195	6.253618485	5.597442421
9.440099647	9.304402078	10.4666169	9.740421109
6.168471946	6.174658045	7.726183812	7.232575149
6.622293242	5.280416631	7.3008678	6.62577442
7.684876023	7.73805901	8.925559837	7.873504371
8.071610564	8.636137564	8.978663547	8.378817045
5.917343536	4.86499514	6.528362151	6.529427696
6.61837815	6.198898662	6.859841266	6.135273454
6.53778129	7.615253922	7.827402134	7.672014061
5.870240981	5.99329829	6.572822639	6.899441427
7.309810911	7.277042392	8.222281471	7.650539328
7.868795077	7.96462787	10.23702834	8.707147033
7.932864676	6.403670902	8.720264903	7.622885615
7.964632403	8.083035113	8.564465274	8.291188452
7.766827105	8.144787081	8.796747278	8.611090279
10.88500946	11.29927327	12.43408217	11.12934195
7.523866088	7.505871865	7.355455649	5.768638026
5.523375769	4.899776882	7.32434432	6.763800617
11.6186457	11.89143586	12.84661801	11.73270037

6.518523109	5.252139808	7.885154797	7.051717246
8.233938236	8.251826197	8.085144998	8.516751788
5.964057062	6.246140446	8.279603072	7.24674511
7.631426123	6.149669887	8.609996948	7.608129159
7.088911994	5.859829826	7.699128468	7.688367305
8.391183445	8.782292111	9.103196339	8.780394355
7.602512505	8.570284889	7.963663087	7.367352037
5.945752263	7.571281624	8.169064216	6.920094966
8.547723591	8.087463147	9.0830683	8.88872445
8.247928931	8.918073697	7.181064153	7.295142034
5.486360144	5.538406561	6.614006189	6.265635554
9.976946018	10.10828479	10.95201069	10.2888392
6.749012006	7.098277849	8.39371565	7.431171951
8.862128182	8.799305041	10.55290354	9.347083025
5.324134916	5.698688355	6.740317768	6.500564498
7.763559334	8.260177732	8.682351039	9.35696926
9.607100485	10.84182763	9.244414481	9.604338577
6.205798072	5.758545642	5.846515598	8.135514014
8.925475394	8.980945687	9.892261805	9.6513396
7.532458272	7.399023012	8.994910308	8.419346269
6.768787382	5.421115171	7.378452631	5.213098686
8.876540642	9.963716886	8.068578129	8.892619914
5.952422607	5.301817414	7.616244735	5.847530033
7.362053129	5.80999442	7.942096664	7.481494134
5.963600284	5.504679604	7.365258732	6.362001216
7.376592636	7.729533723	8.460849143	8.109021885
6.480104817	6.192847227	6.773327918	6.441689385
8.884154499	9.782223122	7.925002374	8.118593754
6.679698616	6.035473371	7.196235523	6.279292636
9.728287894	10.0640451	10.60410202	9.769672534
8.22398318	9.080944872	8.581000184	8.984111252
5.219920033	5.866775417	6.077006188	5.788720398
6.63515656	6.912296885	7.691760974	7.756791652
5.800294497	5.960864417	7.022056091	5.904019223
7.552843022	7.870465837	8.217092604	9.183801644
5.414186852	4.84635688	6.838733351	5.746169991
7.333665756	6.914130137	8.585718196	7.296894465
7.991957809	8.499727485	8.812792174	8.518642872
5.746353547	5.923450178	6.388998437	5.565354156
7.126711795	6.739854438	8.08314158	8.284972046
6.412623549	6.583433907	6.933558268	7.143570952
6.845388367	6.088857917	6.953981298	7.030629547
8.310756189	8.691265893	9.123895456	8.45462593
5.372355549	5.350890015	6.954719289	6.525172242
5.748799777	6.540067971	6.372941959	5.219566923
7.090685689	6.2052601	8.077979951	6.994293846
6.035360606	4.912422444	6.940729635	5.461193043
10.76700145	10.82566302	11.32977856	10.87768931
5.596712953	6.108176917	5.760076098	4.632417298
6.656728402	7.198038734	8.069550344	7.325167378
6.623853267	5.394101578	6.370972424	5.840772086
6.234788025	6.191815301	8.283460501	7.471693108
5.861922875	6.059349169	7.117809228	6.305311574
5.960790289	5.232640886	7.03707487	6.130800294
7.472588486	7.550901114	8.247332896	7.53808213
6.745450263	6.253877499	7.565941783	7.916941503
8.299051419	8.676128611	9.168772602	8.609593992
6.388899618	7.069090973	7.464447839	7.839115979
10.59899716	10.90029622	11.03169799	10.60591127
13.46939356	13.87823259	14.57693001	14.32189277
11.8930159	12.009842	12.47725129	12.05602421
7.399618788	7.160345389	6.441072369	8.201407409

7.213980769	7.817936392	8.555714952	8.394362242
7.931091051	9.021414691	8.316209504	7.937019893
8.740495329	9.058824002	9.275972694	10.94474795
6.965019408	5.970110683	7.679595678	7.399027778
8.51273792	8.855044962	10.45965566	9.612381666
5.310090402	5.145108194	6.331594732	5.997110949
8.766751428	9.418881	9.834807514	9.620682305
6.738673722	5.144070682	6.695350613	7.328092295
8.793979631	8.739389047	9.280300064	8.765699679
9.671518583	10.80693769	9.090201453	9.871562931
10.69765498	11.03200813	11.21247432	11.22868665
9.194967992	10.86209271	8.814965456	9.866623291
7.092650893	7.353874939	8.565355573	8.057224606
5.231837754	4.4476458	6.440823129	5.790545823
6.803448575	5.810833394	6.000670389	5.618358292
6.742320018	7.258776966	7.849080904	7.204166331
10.16113116	10.41350659	10.64122142	10.5448804
12.21349048	12.55969571	13.12253825	12.70435787
6.38265262	5.408206545	7.520210131	7.028419872
7.395949001	6.784645065	9.4024854	8.684292218
6.574644634	5.018186402	7.428036747	5.44872707
7.213939872	6.815031857	9.034105755	8.028853942
9.604521213	8.878258881	10.38521972	9.27996426
6.928974522	6.909774241	8.490865238	8.354798554
8.96844373	8.849112268	9.914680563	9.061774343
7.328757725	7.318938206	8.043487065	7.387381149
9.168379233	9.092323031	9.854350846	9.612845908
5.879517393	5.914554626	7.908402089	7.303071074
7.454343422	6.7160052	8.735239072	7.495910486
6.479522625	6.635914477	7.639247719	6.93370004
5.700717537	4.671525179	6.54096652	6.277072724
7.930067708	8.151534667	9.487728977	8.829524219
7.382448088	7.112182232	7.931528199	7.822475641
7.19219303	7.345474289	8.306760855	8.667530319
7.073241909	6.565592582	8.312707759	8.650749809
6.044702745	5.119922635	7.577176559	6.57693246
9.891336864	9.836934671	11.03662686	10.37674977
10.7393649	11.04666418	11.66612013	10.94129479
8.425058818	9.405966349	6.991269437	8.203137618
6.818023164	7.338953225	7.299693976	8.563012284
8.385310776	8.948663777	8.800304574	9.189161265
10.85092713	11.13049008	11.98277561	11.13901062
10.08191344	10.27105554	11.78688574	10.0820563
5.536037604	5.548094663	7.061309196	6.783208348
5.384437972	5.748769599	6.508977343	7.410069342
6.868736887	7.061320585	6.981769137	5.721883134
6.428864226	6.337131109	7.531861864	6.508285799
7.771106294	6.770403476	8.248408477	9.483832438
7.873774465	7.976162182	8.330944452	8.417637652
6.814205564	7.026719558	8.784373387	7.760429018
8.473105124	8.132157829	9.163825486	8.584284624
5.895204259	6.00475356	6.446657626	6.251618189
5.347028499	5.129976743	5.926191629	5.656894282
8.652882296	9.033629745	9.869513746	10.14336433
8.612297327	9.140608781	9.458467355	9.330401167
8.588196687	8.465402407	10.01765226	9.468774319
7.146167185	7.486404084	8.726411712	8.215582545
5.066902922	5.687355187	7.710238075	5.132680542
6.439103014	5.792292529	7.079039832	7.401082684
6.340698634	6.442440007	7.246834101	7.168008792
5.24303458	5.211765019	6.142850437	6.361924547
5.555306089	5.500194898	6.302010041	5.366262493

7.126848561	6.591458239	8.18663062	6.888686384
7.659375055	7.520549862	8.609992847	8.360439777
5.159812869	4.500684842	5.790576444	5.397269161
5.639911885	4.625147163	6.132725569	5.044171252
7.807340126	8.451383609	8.525527029	8.356954475
8.626391675	9.888491526	8.964966414	8.397945071
6.617863595	5.965463158	7.399177713	6.81097302
6.812517329	7.195476106	6.842042425	6.958069482
7.038969903	6.580308735	8.221958576	6.898934459
7.447147394	7.594813822	8.476786318	8.254202308
7.361633068	7.607169043	8.227093761	7.877700259
7.881403421	8.001857691	9.029201654	8.462970468
6.37784842	6.13306079	7.460220985	7.43479605
6.594753689	6.749417023	7.999398212	7.148618725
5.996550081	5.72951171	7.431424325	6.927150196
6.203765036	5.575971661	5.832258228	5.373795167
7.044059689	7.29358208	8.22388752	7.855684862
5.475872829	5.530687594	6.310009401	5.633419084
6.374575725	6.378928735	6.894373347	6.447349696
8.021961138	7.890793101	9.362728193	8.573842466
6.719674732	6.794095482	7.233025058	5.757999297
9.179174778	10.09970265	8.834423672	8.694355367
5.319672383	5.650339391	6.266730136	6.36351509
6.607157854	6.127872782	7.441871032	6.864042546
6.025057564	6.575799559	7.046706951	7.16849791
5.827094417	5.575688193	7.141603351	6.234922696
9.499067389	10.05219527	10.06377749	9.899939953
6.898778232	6.980653544	8.562300491	7.187552313
10.83462903	11.34380517	11.45334344	11.10281677
6.724868488	5.402875384	7.30142688	6.300596714
9.949878678	9.435668526	10.71757662	10.83513611
8.552481333	8.502150242	8.879895247	8.304796889
7.209413128	7.317914804	8.725702322	6.754251196
7.154486479	6.69937859	7.07659849	6.701111011
9.473453208	9.717586608	10.5800163	9.938098404
6.803366872	6.744911287	7.833992973	6.99062739
8.686783372	10.14897481	8.200285713	8.581277798
10.81238937	10.95467601	11.58618782	11.27108734
7.697845799	7.594150331	8.821516569	7.583834545
6.890189531	7.511616205	7.765607286	8.380500564
9.093715556	9.521310553	10.41064963	8.762182962
7.378902626	5.292852737	8.239448929	7.126839911
8.157393481	8.557692515	8.999557539	9.637523746
4.495078208	4.492406352	6.364236452	6.031227728
9.180189418	8.910419399	9.893967356	8.351769612
7.416890931	6.975169003	7.754230416	6.865436402
6.029166477	5.919445296	7.011606058	7.107467986
6.072422376	6.368050965	7.300353021	7.3513462
5.882087627	7.291165459	9.133993173	7.299405132
6.664469902	6.734579218	7.703883934	7.54852991
6.538346351	6.8943256	8.477766121	7.421275534
8.927222725	8.565106484	9.888799148	8.713299545
7.602386531	8.832477505	9.808228007	9.661868907
9.127000785	9.364672055	10.20296668	9.608640404
6.809392073	6.392248018	8.446841008	7.248610799
7.980052295	8.12681028	8.098040355	8.232540998
9.981239738	10.09974242	10.74097684	10.1376885
9.799575283	9.901757825	10.96231546	10.47379614
6.258835544	6.279468794	8.6098312	5.651563591
8.428282598	8.182205745	9.731831292	8.697596467
6.687926149	5.100013594	8.666535893	7.024484352
7.022343871	7.164767991	8.214205528	7.958833407

7.222140042	8.876449577	6.515315125	5.462485994
8.727375361	9.134333359	9.830490852	8.832709922
9.008678406	10.53777596	9.25892096	9.308334442
7.335711225	7.831809348	7.95630351	7.607390327
10.62445339	10.51861484	11.2176531	11.33235271
7.906795333	8.333702145	7.936995259	7.561490513
7.013017091	7.341994707	7.721527944	7.535628911
6.833869286	6.125022217	8.702549061	6.820233573
5.584492991	4.900527205	6.084153887	5.231831
7.485363326	6.013608043	8.310669578	7.325052573
6.020206036	6.638904785	7.628944	8.143281696
6.343400661	6.223745797	6.703246296	6.53777611
7.933897591	7.0379296	8.04030253	9.238065743
7.034756183	6.302171321	6.51087109	6.347853708
7.710006046	8.145248875	8.792224745	8.585975489
9.026498561	9.453267426	10.254571	9.565471124
4.966338032	6.144439859	6.964109183	8.519006728
9.646817736	10.03592373	10.60049007	10.48559968
7.674222684	6.837233801	8.671971293	8.188771752
6.419272974	6.550575579	8.133781106	6.804698219
5.346439608	5.916902595	7.907390652	6.39603973
8.597024673	8.0758502	7.626108716	7.15317949
11.27282789	11.2795266	11.73607835	11.32554279
7.165887216	8.836621241	6.116017286	7.197820888
8.780128165	9.6877235	8.436599964	8.618366985
9.048223592	8.847479215	10.57964151	9.030590143
7.300866752	7.523618405	8.408380524	8.336778689
7.897805613	8.888661845	8.518723719	9.752451518
7.333738996	7.575238377	8.228300273	8.253405776
5.468763002	5.940253956	6.67305417	6.354426677
7.436362574	8.776209037	9.95625632	7.219572754
11.53458162	11.50022326	12.72023738	11.6956063
6.324968439	5.635351752	7.267885437	7.035280283
8.130772666	7.411821606	8.780766797	8.359759245
5.640737926	6.901900299	7.64196195	5.940448068
7.840746888	7.234291987	8.680408593	7.172496631
6.496467896	7.054362415	6.920394345	6.98189785
9.788334138	9.79366378	10.44798624	9.499362939
6.229908981	6.334267845	6.091379841	6.127029386
5.671144267	5.832152548	7.621265577	6.66933074
9.358769968	9.492623952	10.28484048	9.989782021
6.202353527	7.08601727	7.866402034	6.274156616
5.773580321	5.242735066	6.492494044	6.546421567
6.287183632	6.029293483	6.7968371	7.9394554
4.912453318	5.257139816	5.67388002	5.717075387
5.594474267	5.107025296	6.410248591	6.920668726
6.644182383	5.151464082	6.228825065	6.806702138
6.308003961	5.551503665	7.403690053	6.219364994
5.733945957	6.110766541	7.292939936	6.862463807
12.39385127	12.51998811	13.02416905	12.62081281
5.835847257	5.521783725	6.44715119	5.507994367
8.545111782	7.928457021	9.229189327	8.315724087
7.407186619	7.28929346	8.057256093	7.506833852
7.933819841	8.692771997	9.07901786	10.80568018
6.952610255	6.40430103	8.399188629	8.094131702
6.822837361	7.115974583	8.212111726	6.821997946
8.786193026	9.449234331	11.43063359	9.859253718
4.917578529	5.614325003	7.468984009	5.266421697
5.868062708	5.988585438	6.741235542	5.755653287
5.435969049	5.420871475	5.671547129	5.962868727
6.178919363	6.807815077	7.251513239	6.59381707
9.047755752	10.30179072	7.343111275	10.24987074

4.855677998	4.671299156	5.472088163	4.737014836
5.396002225	6.797395092	6.891904541	7.126515741
7.923651613	8.337382598	8.773625838	8.795676973
8.947675044	10.8580941	8.03478647	10.43410367
9.175443042	11.11755017	8.183691512	10.72893858
4.995775427	5.841677904	6.81439948	5.581776228
8.746317903	8.226450945	9.442008224	8.792251769

Supplemntal Table 6. IPA analysis

**Ingenuity Canonical Pathways**	**-log(p-value)**	**Ratio***
HIPPO signaling	3.21	**0.092**
Heme Degradation	2.56	**0.5**
Epithelial Adherens Junction Signaling	2.24	**0.06**
Germ Cell-Sertoli Cell Junction Signaling	2.22	**0.0559**
Sertoli Cell-Sertoli Cell Junction Signaling	2.14	**0.0543**
SPINK1 Pancreatic Cancer Pathway	2.01	**0.0833**
p53 Signaling	1.96	**0.0631**
Actin Nucleation by ARP-WASP Complex	1.95	**0.0806**
Salvage Pathways of Pyrimidine Deoxyribonucleotides	1.91	**0.25**
Intrinsic Prothrombin Activation Pathway	1.89	**0.0952**
Granulocyte Adhesion and Diapedesis	1.74	**0.0497**
MSP-RON Signaling Pathway	1.69	**0.0694**
Adenine and Adenosine Salvage VI	1.66	**1**
Sorbitol Degradation I	1.66	**1**
Integrin Signaling	1.65	**0.0457**
PPAR Signaling	1.63	**0.0594**
Estrogen Receptor Signaling	1.56	**0.0522**
Insulin Receptor Signaling	1.39	**0.0479**
Glucocorticoid Receptor Signaling	1.39	**0.0377**
GDP-L-fucose Biosynthesis I (from GDP-D-mannose)	1.37	**0.5**
Glycine Degradation (Creatine Biosynthesis)	1.37	**0.5**
Spermine Biosynthesis	1.37	**0.5**
Sulfate Activation for Sulfonation	1.37	**0.5**
Neuroprotective Role of THOP1 in Alzheimer's Disease	1.32	**0.05**
Neuregulin Signaling	1.28	0.0538
PTEN Signaling	1.26	0.0484
Oncostatin M Signaling	1.25	0.075
Agranulocyte Adhesion and Diapedesis	1.21	0.0415
Role of Tissue Factor in Cancer	1.18	0.0462
Apelin Cardiac Fibroblast Signaling Pathway	1.08	0.0909
PAK Signaling	1.07	0.0467
Phenylethylamine Degradation I	1.07	0.25
Glioma Invasiveness Signaling	1.07	0.0526
Chemokine Signaling	1.06	0.0519
Methionine Degradation I (to Homocysteine)	1.05	0.087
GM-CSF Signaling	1.03	0.0506
Glutathione Redox Reactions I	1.02	0.0833
Ephrin Receptor Signaling	1.01	0.0391
Cysteine Biosynthesis III (mammalia)	0.99	0.08
Creatine-phosphate Biosynthesis	0.982	0.2
Protein Citrullination	0.982	0.2
CD27 Signaling in Lymphocytes	0.964	0.0566
Triacylglycerol Degradation	0.945	0.0556
HGF Signaling	0.925	0.042
Macropinocytosis Signaling	0.913	0.046
Rapoport-Luebering Glycolytic Shunt	0.907	0.167
JAK/Stat Signaling	0.887	0.0449
NGF Signaling	0.87	0.0403

Hepatic Cholestasis	0.861	0.0375
Atherosclerosis Signaling	0.839	0.0394
α-Adrenergic Signaling	0.837	0.043
Bladder Cancer Signaling	0.825	0.0426
HER-2 Signaling in Breast Cancer	0.825	0.0426
Communication between Innate and Adaptive Immune Cells	0.802	0.0417
Salvage Pathways of Pyrimidine Ribonucleotides	0.791	0.0412
Phospholipase C Signaling	0.775	0.0327
Netrin Signaling	0.773	0.0462
PXR/RXR Activation	0.773	0.0462
Circadian Rhythm Signaling	0.772	0.0588
Retinoate Biosynthesis I	0.772	0.0588
Coagulation System	0.752	0.0571
Eicosanoid Signaling	0.746	0.0448
Leucine Degradation I	0.745	0.111
Pathogenesis of Multiple Sclerosis	0.745	0.111
Prostanoid Biosynthesis	0.745	0.111
Melanocyte Development and Pigmentation Signaling	0.727	0.0388
STAT3 Pathway	0.727	0.0388
HMGB1 Signaling	0.727	0.036
CNTF Signaling	0.72	0.0435
IL-10 Signaling	0.72	0.0435
ErbB Signaling	0.717	0.0385
Colorectal Cancer Metastasis Signaling	0.717	0.0315
CDK5 Signaling	0.707	0.0381
Superpathway of Methionine Degradation	0.698	0.0526
ERK5 Signaling	0.695	0.0423
Cholecystokinin/Gastrin-mediated Signaling	0.687	0.0374
Inhibition of Matrix Metalloproteases	0.681	0.0513
Ephrin B Signaling	0.672	0.0411
SAPK/JNK Signaling	0.659	0.0364
UVA-Induced MAPK Signaling	0.65	0.036
NF-κB Signaling	0.649	0.0321
Chronic Myeloid Leukemia Signaling	0.641	0.0357
Toll-like Receptor Signaling	0.638	0.0395
Guanosine Nucleotides Degradation III	0.634	0.0833
RAR Activation	0.629	0.0316
Role of Hypercytokinemia/hyperchemokinemia in the Pathogenesis of Influenza	0.618	0.0465
Role of MAPK Signaling in the Pathogenesis of Influenza	0.617	0.0385
VDR/RXR Activation	0.617	0.0385
Cholesterol Biosynthesis I	0.604	0.0769
Cholesterol Biosynthesis II (via 24,25-dihydrolanosterol)	0.604	0.0769
Cholesterol Biosynthesis III (via Desmosterol)	0.604	0.0769
Oleate Biosynthesis II (Animals)	0.604	0.0769
Role of IL-17A in Psoriasis	0.604	0.0769
Urate Biosynthesis/Inosine 5'-phosphate Degradation	0.604	0.0769
γ-glutamyl Cycle	0.604	0.0769
Role of RIG1-like Receptors in Antiviral Innate Immunity	0.604	0.0455
B Cell Receptor Signaling	0.603	0.0309
Telomerase Signaling	0.599	0.0342
Apelin Adipocyte Signaling Pathway	0.596	0.0375
Antiproliferative Role of Somatostatin Receptor 2	0.586	0.037
Paxillin Signaling	0.583	0.0336
3-phosphoinositide Degradation	0.581	0.0316

Colanic Acid Building Blocks Biosynthesis	0.576	0.0714
DNA Double-Strand Break Repair by Homologous Recombination	0.576	0.0714
DNA Double-Strand Break Repair by Non-Homologous End Joining	0.576	0.0714
Phenylalanine Degradation IV (Mammalian, via Side Chain)	0.576	0.0714
GDNF Family Ligand-Receptor Interactions	0.576	0.0366
IL-15 Signaling	0.576	0.0366
Role of IL-17F in Allergic Inflammatory Airway Diseases	0.576	0.0435
Thyroid Cancer Signaling	0.576	0.0435
LXR/RXR Activation	0.567	0.0331
Cardiac Hypertrophy Signaling	0.563	0.029
Angiopoietin Signaling	0.557	0.0357
Gαi Signaling	0.552	0.0325
Telomere Extension by Telomerase	0.55	0.0667
IL-8 Signaling	0.549	0.0296
HIF1α Signaling	0.544	0.0323
Estrogen-Dependent Breast Cancer Signaling	0.539	0.0349
Regulation of IL-2 Expression in Activated and Anergic T Lymphocytes	0.539	0.0349
UVC-Induced MAPK Signaling	0.538	0.0408
Apelin Endothelial Signaling Pathway	0.537	0.032
Axonal Guidance Signaling	0.528	0.0263
Adenosine Nucleotides Degradation II	0.527	0.0625
Chondroitin Sulfate Degradation (Metazoa)	0.527	0.0625
Clathrin-mediated Endocytosis Signaling	0.526	0.029
Endocannabinoid Developing Neuron Pathway	0.523	0.0315
Renal Cell Carcinoma Signaling	0.522	0.0341
Thrombin Signaling	0.51	0.0286
Breast Cancer Regulation by Stathmin1	0.505	0.0284
Leukocyte Extravasation Signaling	0.505	0.0284
Regulation of Actin-based Motility by Rho	0.505	0.0333
Dermatan Sulfate Degradation (Metazoa)	0.505	0.0588
PI3K/AKT Signaling	0.502	0.0308
CXCR4 Signaling	0.499	0.0292
GNRH Signaling	0.499	0.0292
eNOS Signaling	0.493	0.0291
Semaphorin Signaling in Neurons	0.492	0.0377
Fcγ Receptor-mediated Phagocytosis in Macrophages and Monocytes	0.488	0.0326
Differential Regulation of Cytokine Production in Macrophages and T Helper Cells by IL-17A and IL-17F	0.485	0.0556
FLT3 Signaling in Hematopoietic Progenitor Cells	0.481	0.0323
PEDF Signaling	0.481	0.0323
Adipogenesis pathway	0.475	0.0299
IL-6 Signaling	0.475	0.0299
IL-4 Signaling	0.473	0.0319
Cancer Drug Resistance By Drug Efflux	0.471	0.0364
GADD45 Signaling	0.466	0.0526
Histamine Degradation	0.466	0.0526
Purine Nucleotides Degradation II (Aerobic)	0.466	0.0526
CCR3 Signaling in Eosinophils	0.463	0.0294
Apoptosis Signaling	0.458	0.0312
Granzyme A Signaling	0.448	0.05
The Visual Cycle	0.448	0.05

Acute Myeloid Leukemia Signaling	0.443	0.0306
P2Y Purigenic Receptor Signaling Pathway	0.438	0.0286
Oxidative Ethanol Degradation III	0.431	0.0476
PPARα/RXRα Activation	0.418	0.0269
Fatty Acid α-oxidation	0.415	0.0455
D-myo-inositol (1,4,5,6)-Tetrakisphosphate Biosynthesis	0.415	0.0278
D-myo-inositol (3,4,5,6)-tetrakisphosphate Biosynthesis	0.415	0.0278
Melanoma Signaling	0.414	0.0328
autophagy	0.406	0.0323
Differential Regulation of Cytokine Production in Intestinal Epithelial Cells by IL-17A and IL-17F	0.4	0.0435
Putrescine Degradation III	0.4	0.0435
Activation of IRF by Cytosolic Pattern Recognition Receptors	0.397	0.0317
Regulation of Cellular Mechanics by Calpain Protease	0.397	0.0317
CDP-diacylglycerol Biosynthesis I	0.386	0.0417
TCA Cycle II (Eukaryotic)	0.386	0.0417
Hereditary Breast Cancer Signaling	0.383	0.0267
Production of Nitric Oxide and Reactive Oxygen Species in Macrophages	0.38	0.0258
Ethanol Degradation IV	0.373	0.04
IL-17A Signaling in Gastric Cells	0.373	0.04
Tryptophan Degradation X (Mammalian, via Tryptamine)	0.373	0.04
VEGF Signaling	0.371	0.0275
Cell Cycle: G1/S Checkpoint Regulation	0.366	0.0299
Lymphotoxin β Receptor Signaling	0.366	0.0299
IGF-1 Signaling	0.36	0.027
Antiproliferative Role of TOB in T Cell Signaling	0.36	0.0385
Estrogen-mediated S-phase Entry	0.36	0.0385
Gluconeogenesis I	0.36	0.0385
Glycolysis I	0.36	0.0385
Lipid Antigen Presentation by CD1	0.36	0.0385
NAD Salvage Pathway II	0.36	0.0385
Phosphatidylglycerol Biosynthesis II (Non-plastidic)	0.36	0.0385
Remodeling of Epithelial Adherens Junctions	0.351	0.029
3-phosphoinositide Biosynthesis	0.35	0.0249
Gap Junction Signaling	0.35	0.0249
D-myo-inositol (1,4,5)-Trisphosphate Biosynthesis	0.348	0.037
Sirtuin Signaling Pathway	0.344	0.024
Endometrial Cancer Signaling	0.344	0.0286
IL-2 Signaling	0.344	0.0286
ERK/MAPK Signaling	0.338	0.0245
IL-15 Production	0.337	0.0357
Superpathway of Cholesterol Biosynthesis	0.337	0.0357
Thrombopoietin Signaling	0.337	0.0282
mTOR Signaling	0.33	0.0243
Signaling by Rho Family GTPases	0.327	0.0238
Xenobiotic Metabolism Signaling	0.327	0.0236
D-myo-inositol-5-phosphate Metabolism	0.326	0.0247
p38 MAPK Signaling	0.317	0.0252
TNFR2 Signaling	0.315	0.0333
PKCθ Signaling in T Lymphocytes	0.313	0.0242
G Beta Gamma Signaling	0.312	0.025
Glioma Signaling	0.312	0.025
Agrin Interactions at Neuromuscular Junction	0.311	0.0267
ErbB2-ErbB3 Signaling	0.311	0.0267

Myc Mediated Apoptosis Signaling	0.305	0.0263
Tight Junction Signaling	0.305	0.024
Aldosterone Signaling in Epithelial Cells	0.301	0.0238
Dopamine Receptor Signaling	0.299	0.026
Role of JAK1 and JAK3 in γc Cytokine Signaling	0.299	0.026
Rac Signaling	0.298	0.0244
Fatty Acid β-oxidation I	0.296	0.0312
G Protein Signaling Mediated by Tubby	0.296	0.0312
ErbB4 Signaling	0.293	0.0256
Tec Kinase Signaling	0.293	0.0235
SPINK1 General Cancer Pathway	0.287	0.0253
FXR/RXR Activation	0.284	0.0238
Synaptic Long Term Potentiation	0.284	0.0238
Inhibition of Angiogenesis by TSP1	0.278	0.0294
Cyclins and Cell Cycle Regulation	0.276	0.0247
Natural Killer Cell Signaling	0.275	0.0234
Renin-Angiotensin Signaling	0.275	0.0234
fMLP Signaling in Neutrophils	0.271	0.0233
LPS/IL-1 Mediated Inhibition of RXR Function	0.27	0.0224
Acute Phase Response Signaling	0.27	0.0227
BMP signaling pathway	0.27	0.0244
RhoGDI Signaling	0.267	0.0226
Neurotrophin/TRK Signaling	0.265	0.0241
Non-Small Cell Lung Cancer Signaling	0.265	0.0241
Role of NFAT in Cardiac Hypertrophy	0.263	0.0222
FcγRIIB Signaling in B Lymphocytes	0.255	0.0235
Small Cell Lung Cancer Signaling	0.255	0.0235
Dopamine Degradation	0.253	0.027
Ethanol Degradation II	0.253	0.027
Notch Signaling	0.246	0.0263
Erythropoietin Signaling	0.245	0.023
14-3-3-mediated Signaling	0.239	0.0219
Iron homeostasis signaling pathway	0.239	0.0219
Role of Pattern Recognition Receptors in Recognition of Bacteria and Viruses	0.239	0.0219
IL-3 Signaling	0.236	0.0225
Prolactin Signaling	0.236	0.0225
Noradrenaline and Adrenaline Degradation	0.232	0.025
Altered T Cell and B Cell Signaling in Rheumatoid Arthritis	0.231	0.0222
IL-17 Signaling	0.227	0.022
Mechanisms of Viral Exit from Host Cells	0.225	0.0244
Retinol Biosynthesis	0.219	0.0238
Serotonin Receptor Signaling	0.213	0.0233
Stearate Biosynthesis I (Animals)	0.207	0.0227
IL-9 Signaling	0.201	0.0222
iNOS Signaling	0.201	0.0222
AMPK Signaling	0	0.0139
ATM Signaling	0	0.0102
Actin Cytoskeleton Signaling	0	0.0215
Adrenomedullin signaling pathway	0	0.02
Amyloid Processing	0	0.0196
Amyotrophic Lateral Sclerosis Signaling	0	0.00901
Androgen Signaling	0	0.0146
Antioxidant Action of Vitamin C	0	0.00926
Aryl Hydrocarbon Receptor Signaling	0	0.0211
CCR5 Signaling in Macrophages	0	0.0211

CD40 Signaling	0	0.0127
CREB Signaling in Neurons	0	0.0183
Calcium Signaling	0	0.0194
Cardiac β-adrenergic Signaling	0	0.0213
Caveolar-mediated Endocytosis Signaling	0	0.0141
Cdc42 Signaling	0	0.018
Cell Cycle: G2/M DNA Damage Checkpoint Regulation	0	0.02
Cellular Effects of Sildenafil (Viagra)	0	0.0153
Ceramide Signaling	0	0.0202
Corticotropin Releasing Hormone Signaling	0	0.00719
Death Receptor Signaling	0	0.0215
Dendritic Cell Maturation	0	0.00513
Docosahexaenoic Acid (DHA) Signaling	0	0.0192
Dopamine-DARPP32 Feedback in cAMP Signaling	0	0.0183
EIF2 Signaling	0	0.00881
Endocannabinoid Cancer Inhibition Pathway	0	0.00637
Endothelin-1 Signaling	0	0.0204
FAK Signaling	0	0.019
Fc Epsilon RI Signaling	0	0.016
G-Protein Coupled Receptor Signaling	0	0.0106
GABA Receptor Signaling	0	0.0211
GP6 Signaling Pathway	0	0.0149
GPCR-Mediated Integration of Enteroendocrine Signaling Exemplified by an L Cell	0	0.0137
GPCR-Mediated Nutrient Sensing in Enteroendocrine Cells	0	0.0179
Glioblastoma Multiforme Signaling	0	0.0179
Graft-versus-Host Disease Signaling	0	0.0208
Growth Hormone Signaling	0	0.0119
Gustation Pathway	0	0.0195
Gα12/13 Signaling	0	0.0142
Gαq Signaling	0	0.0124
Gαs Signaling	0	0.0182
Heparan Sulfate Biosynthesis	0	0.012
Heparan Sulfate Biosynthesis (Late Stages)	0	0.0132
Hepatic Fibrosis / Hepatic Stellate Cell Activation	0	0.016
Huntington's Disease Signaling	0	0.02
Hypoxia Signaling in the Cardiovascular System	0	0.0133
IL-1 Signaling	0	0.0217
IL-12 Signaling and Production in Macrophages	0	0.0137
IL-17A Signaling in Airway Cells	0	0.0128
ILK Signaling	0	0.0203
Induction of Apoptosis by HIV1	0	0.0164
LPS-stimulated MAPK Signaling	0	0.0215
Leptin Signaling in Obesity	0	0.0116
Mitochondrial Dysfunction	0	0.0117
Mitotic Roles of Polo-Like Kinase	0	0.0152
Molecular Mechanisms of Cancer	0	0.0203
Mouse Embryonic Stem Cell Pluripotency	0	0.0179
NF-κB Activation by Viruses	0	0.0215
NRF2-mediated Oxidative Stress Response	0	0.015
Neuroinflammation Signaling Pathway	0	0.00965
Nitric Oxide Signaling in the Cardiovascular System	0	0.00885
OX40 Signaling Pathway	0	0.011
Opioid Signaling Pathway	0	0.0203
Osteoarthritis Pathway	0	0.0189
Ovarian Cancer Signaling	0	0.0134

Oxidative Phosphorylation	0	0.0183
PDGF Signaling	0	0.0208
PI3K Signaling in B Lymphocytes	0	0.0147
Pancreatic Adenocarcinoma Signaling	0	0.0167
Phagosome Formation	0	0.0152
Phagosome Maturation	0	0.0203
Phospholipases	0	0.0161
Prostate Cancer Signaling	0	0.0194
Protein Kinase A Signaling	0	0.0174
Protein Ubiquitination Pathway	0	0.0189
Pyridoxal 5'-phosphate Salvage Pathway	0	0.0154
RANK Signaling in Osteoclasts	0	0.0196
Reelin Signaling in Neurons	0	0.0217
Regulation of eIF4 and p70S6K Signaling	0	0.0123
Regulation of the Epithelial-Mesenchymal Transition Pathway	0	0.0154
Relaxin Signaling	0	0.0127
Retinoic acid Mediated Apoptosis Signaling	0	0.0161
RhoA Signaling	0	0.0161
Role of BRCA1 in DNA Damage Response	0	0.0125
Role of CHK Proteins in Cell Cycle Checkpoint Control	0	0.0175
Role of Cytokines in Mediating Communication between Immune Cells	0	0.0185
Role of IL-17A in Arthritis	0	0.0145
Role of Macrophages, Fibroblasts and Endothelial Cells in Rheumatoid Arthritis	0	0.0156
Role of NANOG in Mammalian Embryonic Stem Cell Pluripotency	0	0.0156
Role of NFAT in Regulation of the Immune Response	0	0.0208
Role of Osteoblasts, Osteoclasts and Chondrocytes in Rheumatoid Arthritis	0	0.00858
Role of Wnt/GSK-3β Signaling in the Pathogenesis of Influenza	0	0.013
Serotonin Degradation	0	0.013
Sperm Motility	0	0.0157
Sphingosine-1-phosphate Signaling	0	0.016
Sumoylation Pathway	0	0.0104
Superpathway of Inositol Phosphate Compounds	0	0.0212
Synaptic Long Term Depression	0	0.0167
Systemic Lupus Erythematosus Signaling	0	0.0214
T Cell Receptor Signaling	0	0.0174
TGF-β Signaling	0	0.0215
TNFR1 Signaling	0	0.02
TR/RXR Activation	0	0.0102
TREM1 Signaling	0	0.0133
Th17 Activation Pathway	0	0.011
Transcriptional Regulatory Network in Embryonic Stem Cells	0	0.0185
Triacylglycerol Biosynthesis	0	0.0217
Type II Diabetes Mellitus Signaling	0	0.00649
Unfolded protein response	0	0.0179
VEGF Family Ligand-Receptor Interactions	0	0.0213
Virus Entry via Endocytic Pathways	0	0.0172
Wnt/β-catenin Signaling	0	0.0174
cAMP-mediated signaling	0	0.00439
p70S6K Signaling	0	0.0145

*Ratio = Number of pathway genes in list / Total number of genes in pathway

Molecules
CSNK1E,TEAD3,PARD3,PPP1R3C,TEAD1,WWTR1,WWC1,DLG5
HMOX2,BLVRB
EPN3,PARD3,TUBB6,SORBS1,MAGI1,RAP2B,WASL,YES1,RAP2A
EPN3,PLS1,TUBB6,SORBS1,RHOB,RAP2B,MAP3K4,WASL,MAP3K9,RAP2A
EPN3,PLS1,TUBB6,SORBS1,CLDN12,RAP2B,CLDN1,MAP3K4,MAP3K9,RAP2A
CPA4,KLK13,KLK11,KLK10,KLK7
KAT2B,PERP,JMY,PPP1R13B,BCL2L1,THBS1,PMAIP1
NCK2,RHOB,RAP2B,WASL,RAP2A
CDA,APOBEC3A
KLK13,KLK11,KLK10,KLK7
CCL4L1/CCL4L2,MMP25,IL18RAP,CLDN12,CCL20,CLDN1,CCL4,CCL3L3,IL36A
RPS6KA2,KLK13,KLK11,KLK10,KLK7
ADK,
SORD,
TSPAN6,NCK2,RHOB,RAP2B,LIMS1,WASL,ARF6,MPRIP,GRB7,RAP2A
PPARD,IL18RAP,RAP2B,IL36A,CITED2,RAP2A
KAT2B,CCNC,MED21,RAP2B,MED18,ESR1,RAP2A
SCNN1G,PPP1R3C,TRIP10,SCNN1B,PTPRF,RAP2B,RAP2A
KAT2B,KRT78,KRT4,HSPA1A/HSPA1B,KRT18,KRT6A,KRT80,RAP2B,BCL2L1,KRT16,KRT15,ESR1,RAP2A
GMDS,
GATM,
SMS,
PAPSS2,
PRSS27,PRSS8,MME,KLK11,KLK10,KLK7
RAP2B,ERRFI1,EREG,GRB7,RAP2A
MAGI1,CNKSR3,RAP2B,BCL2L1,MAGI3,RAP2A
EPAS1,RAP2B,RAP2A
CCL4L1/CCL4L2,MMP25,CLDN12,CCL20,CLDN1,CCL4,CCL3L3,IL36A
RPS6KA2,CTGF,RAP2B,BCL2L1,YES1,RAP2A
ACE2,CTGF
ARHGAP10,NCK2,RAP2B,WASL,RAP2A
ALDH3A2,
RHOB,RAP2B,TIMP2,RAP2A
RAP2B,CCL4,MPRIP,RAP2A
PRMT5,AHCY
RAP2B,BCL2L1,CISH,RAP2A
PRDX6,GPX3
EFNB2,NCK2,SORBS1,GNG12,RAP2B,WASL,RAP2A
PRMT5,AHCY
CKB,
PADI1,
BCL2L1,MAP3K4,MAP3K9
PRDX6,ABHD12,MGLL
ELK3,RAP2B,MAP3K4,MAP3K9,RAP2A
USP6NL,RAP2B,ARF6,RAP2A
BPGM,
RAP2B,BCL2L1,CISH,RAP2A
RPS6KA2,RAP2B,MAP3K4,MAP3K9,RAP2A

IL18RAP,MAP3K4,ATP8B1,IL36A,ESR1,ADCY9
ALOX12,TNFSF14,PCYOX1,PNPLA8,IL36A
GNG12,RAP2B,ADCY9,RAP2A
MMP25,RAP2B,THBS1,RAP2A
PARD3,PARD6B,RAP2B,RAP2A
TLR8,CCL4,CCL3L3,IL36A
CDA,APOBEC3A,UPP1,MAP3K9
PEBP1,RHOB,GNG12,RAP2B,HDAC4,ADCY9,MPRIP,RAP2A
ABLIM3,NCK2,ABLIM1
ALDH3A2,SCD,PAPSS2
CSNK1E,NR1D1
RDH13,SDR16C5
BDKRB2,PLAT
ALOX12,PNPLA8,PTGES
ACADM,
CCL4,
PTGES,
RPS6KA2,RAP2B,ADCY9,RAP2A
RAP2B,CISH,MAP3K9,RAP2A
KAT2B,RHOB,RAP2B,PLAT,RAP2A
RPS6KA2,RAP2B,RAP2A
IL18RAP,BLVRB,IL36A
NCK2,RAP2B,EREG,RAP2A
MMP25,TLR8,RHOB,GNG12,RAP2B,BCL2L1,ADCY9,RAP2A
PPP1R3C,RAP2B,ADCY9,RAP2A
PRMT5,AHCY
RPS6KA2,RAP2B,RAP2A
RHOB,RAP2B,IL36A,RAP2A
MMP25,TIMP2
EFNB2,NCK2,GNG12
RAP2B,MAP3K4,MAP3K9,RAP2A
RPS6KA2,RAP2B,BCL2L1,RAP2A
TLR8,TANK,TAB3,RAP2B,IL36A,RAP2A
RAP2B,BCL2L1,HDAC4,RAP2A
TLR8,TICAM1,IL36A
NT5C3A,
KAT2B,RDH13,SDR16C5,RARG,ADCY9,CITED2
CCL4,IL36A
PNPLA8,RAP2B,RAP2A
PPARD,CCNC,KLF4
DHCR24,
DHCR24,
DHCR24,
SCD,
CCL20,
NT5C3A,
CHAC1,
TANK,DDX58
APBB1IP,RAP2B,BCL2L1,MAP3K4,MAP3K9,RAP2A
ELK3,RAP2B,HDAC4,RAP2A
PRDX6,GPX3,ADCY9
GNG12,RAP2B,RAP2A
NCK2,RAP2B,ARF6,RAP2A
MTMR1,PPTC7,DUSP14,PTPRF,PPP1R13B

GMDS,
NBN,
NBN,
ALDH3A2,
DOK3,RAP2B,RAP2A
RAP2B,BCL2L1,RAP2A
RPS6KA2,CCL4
RAP2B,RAP2A
SCD,IL18RAP,PCYOX1,IL36A
RHOB,GNG12,RAP2B,MAP3K4,MAP3K9,ADCY9,RAP2A
RAP2B,GRB7,RAP2A
GNG12,RAP2B,ADCY9,RAP2A
NBN,
RHOB,GNG12,CSTB,RAP2B,BCL2L1,RAP2A
MMP25,EGLN3,RAP2B,RAP2A
RAP2B,ESR1,RAP2A
TOB1,RAP2B,RAP2A
RAP2B,RAP2A
RAP2B,HDAC4,ADCY9,RAP2A
EFNB2,ABLIM3,MME,TUBB6,NCK2,SEMA7A,GNG12,ABLIM1,RAP2B,WASL,SRGAP1,RAP2A
NT5C3A,
GM2A,
SH3GL1,SNX9,HIP1R,PCYOX1,WASL,ARF6
RAP2B,MGLL,ADCY9,RAP2A
EGLN3,RAP2B,RAP2A
RHOB,GNG12,RAP2B,ADCY9,MPRIP,RAP2A
PPP1R3C,TUBB6,GNG12,RAP2B,ADCY9,RAP2A
MMP25,CLDN12,CLDN1,MAP3K4,WASL,TIMP2
RHOB,WASL,MPRIP
GM2A,
RAP2B,BCL2L1,LIMS1,RAP2A
RHOB,GNG12,RAP2B,ADCY9,RAP2A
RAP2B,MAP3K4,MAP3K9,ADCY9,RAP2A
HSPA1A/HSPA1B,BDKRB2,AQP3,ESR1,ADCY9
RHOB,SEMA7A
NCK2,YES1,ARF6
CCL4,
RPS6KA2,RAP2B,RAP2A
RAP2B,BCL2L1,RAP2A
KAT2B,LPIN1,NR1D2,HDAC4
IL18RAP,RAP2B,IL36A,RAP2A
HMGA1,RAP2B,RAP2A
RAP2B,RAP2A
MAP3K4,
ALDH3A2,
NT5C3A,
GNG12,RAP2B,MPRIP,RAP2A
RAP2B,BCL2L1,RAP2A
H1F0,
RDH13,

PIM2,RAP2B,RAP2A
GNG12,RAP2B,ADCY9,RAP2A
ALDH3A2,
IL18RAP,ADIPOR2,RAP2B,ADCY9,RAP2A
ALDH3A2,
PPTC7,DUSP14,PTPRF,PPP1R13B
PPTC7,DUSP14,PTPRF,PPP1R13B
RAP2B,RAP2A
ULK1,ATG9B
CCL4,
ALDH3A2,
TANK,DDX58
RAP2B,RAP2A
CDS1,
SDHA,
RAP2B,HDAC4,RAP2A,NBN
PPP1R3C,RHOB,PCYOX1,MAP3K4,MAP3K9
ALDH3A2,
CCL20,
ALDH3A2,
RAP2B,BCL2L1,RAP2A
CDKN2B,HDAC4
TNFSF14,BCL2L1
CTGF,RAP2B,RAP2A
TOB1,
ESR1,
BPGM,
BPGM,
ARF6,
NT5C3A,
CDS1,
TUBB6,ARF6
PI4K2A,PPTC7,DUSP14,PTPRF,PPP1R13B
CSNK1E,TUBB6,RAP2B,ADCY9,RAP2A
PI4K2A,
BPGM,SDHA,EPAS1,NDUFB11,H1F0,ATG9B,NBN
RAP2B,RAP2A
RAP2B,RAP2A
PPP1R3C,ELK3,RAP2B,ESR1,RAP2A
PTK6,
DHCR24,
RAP2B,RAP2A
ULK1,RPS6KA2,RHOB,RAP2B,RAP2A
PARD3,RHOB,SEPT10,GNG12,WASL,MAP3K9
ALDH3A2,RAP2B,HDAC4,MAP3K4,MAP3K9,CITED2,RAP2A
PPTC7,DUSP14,PTPRF,PPP1R13B
RPS6KA2,IL18RAP,IL36A
TANK,
RAP2B,MAP3K4,MAP3K9,RAP2A
GNG12,RAP2B,RAP2A
CDKN2B,RAP2B,RAP2A
RAP2B,RAP2A
RAP2B,RAP2A

RAP2B,RAP2A
MPP5,CLDN12,CNKSR3,CLDN1
HSPA1A/HSPA1B,SCNN1G,SCNN1B,AHCY
PPP1R3C,ADCY9
RAP2B,RAP2A
PARD3,RAP2B,RAP2A
ACADM,
GNG12,
RAP2B,RAP2A
RHOB,TNFRSF21,GNG12,YES1
RAP2B,RAP2A
PCYOX1,SCARB1,IL36A
PPP1R3C,RAP2B,RAP2A
THBS1,
CDKN2B,HDAC4
RAP2B,RAP2A,SH2D1B
RAP2B,ADCY9,RAP2A
GNG12,RAP2B,RAP2A
ALDH3A2,IL18RAP,PAPSS2,SCARB1,IL36A
HMOX2,RAP2B,IL36A,RAP2A
RAP2B,RAP2A
RHOB,GNG12,WASL,ESR1
RAP2B,RAP2A
RAP2B,RAP2A
GNG12,RAP2B,HDAC4,ADCY9,RAP2A
RAP2B,RAP2A
CDKN2B,BCL2L1
ALDH3A2,
ALDH3A2,
DTX2,
RAP2B,RAP2A
TUBB6,RAP2B,RAP2A
ATP6V1C2,HMOX2,EPAS1
TLR8,TICAM1,DDX58
RAP2B,RAP2A
RAP2B,RAP2A
ALDH3A2,
TLR8,IL36A
RAP2B,RAP2A
SH3GL1,
RDH13,
ADCY9,
DHCR24,
CISH,
HMGA1,
ULK1,KAT2B,PFKFB2
NBN,
GNG12,RAP2B,WASL,MPRIP,RAP2A
RAP2B,IL36A,ADCY9,RAP2A
CSNK1E,
BCL2L1,
KAT2B,GNG12
PNPLA8,
ALDH3A2,RARG,ESR1
GNG12,CCL4

TANK,
GNG12,RAP2B,ADCY9,RAP2A
MCU,RAP2B,HDAC4,RAP2A
PPP1R3C,GNG12,ADCY9
CD48,
PARD3,WASL,MPRIP
KAT2B,
ADCY9,MPRIP
RAP2B,RAP2A
ADCY9,
TANK,TNFRSF21
IL36A,
BCL2L1,
CSNK1E,PPP1R3C,ADCY9
RAP2B,RAP2A
ADCY9,
PNPLA8,RAP2B,ADCY9,RAP2A
RAP2B,RAP2A
RAP2B,RAP2A
RAP2B,ADCY9,RAP2A
GABRP,ADCY9
LAMC2,APBB1IP
ADCY9,
GNG12,ADCY9
RHOB,RAP2B,RAP2A
IL36A,
RPS6KA2,
SCNN1G,SCNN1B,ADCY9
RAP2B,RAP2A
RHOB,GNG12
GNG12,ADCY9
PRDX6,
PRDX6,
IL18RAP,CTGF,TIMP2
HSPA1A/HSPA1B,SDHA,GNG12,BCL2L1,HDAC4
UBE2G1,
GNG12,ADCY9
ALOX12,PCYOX1
CCL20,
NCK2,RHOB,LIMS1,KRT18
BCL2L1,
RAP2B,RAP2A
ADCY9,
SDHA,NDUFB11
ESPL1,
RHOB,CDKN2B,RAP2B,BCL2L1,PMAIP1,ADCY9,RAP2A,NBN
RAP2B,RAP2A
RAP2B,RAP2A
SCARB1,RAP2B,RAP2A
TLR8,GABRP,TICAM1
BDKRB2,
BCL2L1,
RPS6KA2,RAP2B,YES1,ADCY9,RAP2A
PPARD,IL18RAP,EPAS1,HDAC4
RAP2B,RAP2A

SDHA,NDUFB11
RAP2B,RAP2A
RAP2B,RAP2A
CDKN2B,BCL2L1
TLR8,RHOB
PRDX6,ATP6V1C2,TUBB6
PNPLA8,
RAP2B,RAP2A
PTPN14,PPP1R3C,GNG12,PTPRF,PTPN3,H1F0,ADCY9
USP46,HSPA1A/HSPA1B,USP38,UBE2G1,USP53
MAP3K9,
MAP3K4,MAP3K9
YES1,MAP3K9
RAP2B,RAP2A
PARD6B,RAP2B,RAP2A
GNG12,ADCY9
RARG,
SEPT10,MPRIP
NBN,
NBN,
IL36A,
CCL20,
TLR8,IL18RAP,RAP2B,IL36A,RAP2A
RAP2B,RAP2A
CSNK1E,GNG12,RAP2B,RAP2A
IL18RAP,IL36A
CSNK1E,
ALDH3A2,
PTK6,PNPLA8
RHOB,ADCY9
RHOB,
PI4K2A,PPTC7,DUSP14,PTPRF,PPP1R13B
PNPLA8,RAP2B,RAP2A
PIM2,RAP2B,BDKRB2,IL36A,RAP2A
RAP2B,RAP2A
RAP2B,RAP2A
TANK,
SCARB1,
TLR8,
CCL20,
MEIS1,
LPIN1,
ADIPOR2,
HSPA1A/HSPA1B,
RAP2B,RAP2A
RAP2B,RAP2A
CSNK1E,PPARD,RARG
ADCY9,
RAP2B,RAP2A

Supplementary Table 7. SLIMM Summary

Genus	Patient #1		Patient #2		Patient #3		Patie
	Reads	Abundance (%)	Reads	Abundance (%)	Reads	Abundance (%)	Reads
Lactobacillus	496988	5.336	27628410	85.533	36187384	89.966	2351073
Gardnerella	2283604	24.517	888996	2.752	739328	1.838	9571846
Prevotella	2266106	24.329	12442	0.039	329207	0.818	3120823
Atopobium	976342	10.482	12449	0.039	243979	0.607	1115467
Mobiluncus	181048	1.944	721	0.002	603	0.001	641
Streptococcus	1349	0.014	2890910	8.95	239444	0.595	1975
Peptoniphilus	272210	2.922	58063	0.18	29881	0.074	406207
Sneathia	58667	0.63	7256	0.022	12008	0.03	144785
Mageeibacillus	1648	0.018	1575	0.005	11841	0.029	37424
Aerococcus	95115	1.021	24213	0.075	21946	0.055	155190
Anaerococcus	9818	0.105	949	0.003	24647	0.061	66261
Mycoplasma	264319	2.838	1091	0.003	10419	0.026	157140
Peptostreptococcus	77303	0.83	461	0.001	220	0.001	1064
Gemella	74529	0.8	13807	0.043	16610	0.041	138085
Planococcus	0	0	0	0	311633	0.775	6712
Porphyromonas	3682	0.04	0	0	0	0	130
Fusobacterium	33409	0.359	0	0	0	0	0
Dialister	55018	0.591	601	0.002	12029	0.03	89611
Bifidobacterium	20925	0.225	3085	0.01	5070	0.013	90329
Veillonella	97	0.001	142279	0.44	581	0.001	400
Megasphaera	31579	0.339	519	0.002	9881	0.025	55129
Gemmata	18520	0.199	0	0	21513	0.053	5546
Arcanobacterium	38484	0.413	256	0.001	6636	0.016	65252
Alloscardovia	5507	0.059	912	0.003	3110	0.008	44359
Necropsobacter	0	0	0	0	81080	0.202	98
Ureaplasma	1131	0.012	17773	0.055	3719	0.009	169
Parvimonas	2865	0.031	60	<0.001	956	0.002	8524
Acinetobacter	5690	0.061	0	0	11027	0.027	440
Nocardia	3384	0.036	9510	0.029	2599	0.006	942
Curtobacterium	0	0	0	0	10670	0.027	3254
Finegoldia	154	0.002	2023	0.006	138	<0.001	24
Microbacterium	1048	0.011	0	0	7479	0.019	681
Butyrivibrio	0	0	0	0	1139	0.003	23115
Campylobacter	0	0	0	0	82	<0.001	0
Clostridium	0	0	0	0	1507	0.004	18176
Paeniglutamicibacter	3326	0.036	0	0	473	0.001	501
Rhodococcus	0	0	0	0	940	0.002	2808
Eubacterium	0	0	0	0	903	0.002	12480
Stomatobaculum	0	0	0	0	187	<0.001	646
Beggiatoa	0	0	0	0	1687	0.004	374
Achromobacter	0	0	0	0	9051	0.023	1338
Roseburia	0	0	0	0	0	0	10540
Bacteroides	1429	0.015	0	0	131	<0.001	2742
Lachnoanaerobaculum	0	0	0	0	411	0.001	5055
Lachnobacterium	0	0	0	0	792	0.002	5654
Vibrio	714	0.008	0	0	1977	0.005	280
Alloprevotella	3605	0.039	0	0	27	<0.001	519
Actinomyces	75	0.001	284	0.001	145	<0.001	558
Fenollaria	0	0	0	0	0	0	57
Pseudomonas	0	0	0	0	1090	0.003	0
Corynebacterium	0	0	3481	0.011	166	<0.001	358
Scardovia	617	0.007	115	<0.001	62	<0.001	1304
Pseudobutyrivibrio	0	0	0	0	432	0.001	3441
Shuttleworthia	0	0	0	0	413	0.001	3324
Bulleidia	580	0.006	0	0	162	<0.001	1408
Barnesiella	0	0	0	0	0	0	0
Oleiphilus	0	0	0	0	2405	0.006	0
Staphylococcus	0	0	2245	0.007	999	0.002	88
Chlamydia	2944	0.032	0	0	0	0	0

Varibaculum	0	0	128	<0.001	0	0	0
Blautia	0	0	0	0	0	0	2604
Xanthomonas	0	0	0	0	867	0.002	248
Haemophilus	0	0	1860	0.006	80	<0.001	0
Olsenella	178	0.002	0	0	26	<0.001	1013
Sutterella	1394	0.015	0	0	0	0	0
Pedobacter	0	0	0	0	1267	0.003	0
Facklamia	0	0	1405	0.004	0	0	152
Hallella	1612	0.017	0	0	0	0	97
Ruminococcus	0	0	0	0	0	0	1907
Achromatium	473	0.005	0	0	338	0.001	87
Lachnospira	0	0	0	0	212	0.001	1408
Sanguibacteroides	0	0	0	0	981	0.002	0
Streptomyces	0	0	0	0	772	0.002	0
Catonella	0	0	0	0	136	<0.001	1405
Dorea	0	0	0	0	0	0	1535
Oribacterium	0	0	0	0	116	<0.001	577
Marvinbryantia	0	0	0	0	0	0	1445
Actinobaculum	0	0	455	0.001	29	<0.001	0
Neisseria	0	0	0	0	170	<0.001	1019
Solobacterium	74	0.001	0	0	50	<0.001	526
Levyella	0	0	114	<0.001	0	0	83
Georgenia	0	0	0	0	954	0.002	0
Terrabacter	0	0	0	0	363	0.001	0
Gordonibacter	0	0	0	0	0	0	0
Caulobacter	0	0	0	0	760	0.002	0
Arthrobacter	0	0	0	0	347	0.001	0
Actinotignum	0	0	0	0	0	0	102
Lawsonella	100	0.001	148	<0.001	33	<0.001	220
Kallipyga	0	0	0	0	26	<0.001	141
Collinsella	0	0	0	0	0	0	317
Granulicatella	0	0	502	0.002	0	0	0
Carnobacterium	0	0	0	0	80	<0.001	465
Tannerella	0	0	0	0	0	0	0
Paenibacillus	0	0	0	0	538	0.001	0
Mannheimia	0	0	0	0	517	0.001	0
Johnsonella	0	0	0	0	0	0	511
Oligella	0	0	486	0.002	0	0	0
Streptobacillus	54	0.001	0	0	32	<0.001	379
Caviibacter	123	0.001	0	0	22	<0.001	235
Eremococcus	0	0	69	<0.001	306	0.001	0
Leptotrichia	0	0	0	0	60	<0.001	361
Methanobrevibacter	0	0	0	0	188	<0.001	0
Holdemanella	0	0	0	0	0	0	375
Lachnoclostridium	0	0	0	0	0	0	85
Pseudoalteromonas	0	0	0	0	164	<0.001	0
Massilia	0	0	0	0	255	0.001	0
Burkholderia	0	0	0	0	0	0	240
Filifactor	0	0	0	0	0	0	218
Leuconostoc	33	<0.001	0	0	35	<0.001	0
Thermoanaerobacter	0	0	0	0	205	0.001	0
Rothia	0	0	200	0.001	0	0	0
Alcanivorax	0	0	0	0	102	<0.001	0
Peptoclostridium	0	0	0	0	0	0	180
Candidatus Zinderia	0	0	0	0	0	0	88
Allofustis	0	0	121	<0.001	0	0	38
Paraprevotella	0	0	0	0	0	0	156
Eggerthia	0	0	0	0	0	0	45
Bergeyella	0	0	0	0	74	<0.001	0
Dehalococcoides	53	0.001	0	0	0	0	0
Treponema	0	0	0	0	0	0	128
Pseudoglutamicibacter	0	0	120	<0.001	0	0	0

Parascardovia	0	0	0	0	23	<0.001	96
Pelomonas	0	0	0	0	0	0	113
Phocaeicola	0	0	0	0	0	0	48
Alistipes	0	0	0	0	0	0	108
Cyanothece	0	0	0	0	100	<0.001	0
Defluviitalea	0	0	0	0	0	0	99
Globicatella	0	0	96	<0.001	0	0	0
Odoribacter	0	0	0	0	0	0	81
Cryptobacterium	0	0	0	0	0	0	79
Polaribacter	0	0	0	0	0	0	75
Candidatus Saccharimonas	0	0	0	0	0	0	0
Mogibacterium	0	0	0	0	0	0	73
Helcococcus	0	0	0	0	70	<0.001	0
Prochlorococcus	0	0	0	0	58	<0.001	0
Pseudoramibacter	0	0	0	0	0	0	69
Eggerthella	0	0	0	0	0	0	65
Mitsuokella	0	0	0	0	0	0	52
Blattabacterium	0	0	0	0	0	0	47
Epulopiscium	0	0	0	0	0	0	45
Candidatus Azobacteroides	0	0	0	0	0	0	44
Apibacter	0	0	0	0	0	0	36
Conchiformibius	0	0	0	0	0	0	0
Candidatus Caldatribacteirum	0	0	0	0	32	<0.001	0
Slackia	0	0	0	0	0	0	32
Heliobacterium	0	0	0	0	0	0	30
Weissella	0	0	0	0	24	<0.001	0
Murdochiella	0	0	0	0	20	<0.001	0
Dolosigranulum	0	0	0	0	0	0	0

nt #4	Patient #5		Patient #6		Patient #7		Patie
Abundance (%)	Reads	Abundance (%)	Reads	Abundance (%)	Reads	Abundance (%)	Reads
9.528	3751964	58.019	219765	9.383	72576	0.248	11137246
38.791	1785242	27.606	552906	23.607	5907243	20.2	5366522
12.648	4286	0.066	342812	14.637	7439665	25.44	739
4.521	234426	3.625	25952	1.108	229585	0.785	3168
0.003	1944	0.03	243	0.01	7083337	24.221	332
0.008	703	0.011	5582	0.238	254568	0.87	542181
1.646	1299	0.02	8799	0.376	1083736	3.706	0
0.587	643	0.01	52577	2.245	756005	2.585	69
0.152	438	0.007	156	0.007	709362	2.426	691
0.629	62338	0.964	1079	0.046	13755	0.047	202574
0.269	892	0.014	360	0.015	654587	2.238	221
0.637	152	0.002	74594	3.185	3655	0.012	172588
0.004	0	0	230226	9.83	209043	0.715	219
0.56	402	0.006	16020	0.684	683	0.002	39682
0.027	23688	0.366	0	0	0	0	0
0.001	0	0	5260	0.225	288134	0.985	0
0	0	0	34612	1.478	222954	0.762	0
0.363	1575	0.024	7980	0.341	31062	0.106	4797
0.366	9408	0.145	3850	0.164	33014	0.113	14105
0.002	0	0	319	0.014	2733	0.009	23398
0.223	21	<0.001	7831	0.334	22736	0.078	89
0.022	15184	0.235	13759	0.587	23422	0.08	0
0.264	458	0.007	123	0.005	17012	0.058	295
0.18	2345	0.036	186	0.008	48950	0.167	11126
<0.001	0	0	0	0	0	0	0
0.001	9537	0.147	3210	0.137	0	0	32745
0.035	20	<0.001	40573	1.732	9463	0.032	126
0.002	7719	0.119	5786	0.247	0	0	6909
0.004	1720	0.027	1987	0.085	0	0	10712
0.013	7276	0.113	0	0	0	0	0
<0.001	195	0.003	27	0.001	24233	0.083	70
0.003	5497	0.085	994	0.042	8618	0.029	1287
0.094	0	0	0	0	0	0	0
0	24	<0.001	0	0	21717	0.074	0
0.074	49	0.001	0	0	0	0	0
0.002	1001	0.015	3235	0.138	0	0	7810
0.011	724	0.011	3276	0.14	0	0	0
0.051	0	0	36	0.002	1487	0.005	0
0.003	0	0	0	0	13611	0.047	0
0.002	5185	0.08	3338	0.143	0	0	0
0.005	2407	0.037	0	0	0	0	0
0.043	0	0	0	0	0	0	0
0.011	0	0	0	0	5751	0.02	0
0.02	0	0	0	0	2196	0.008	0
0.023	0	0	0	0	1433	0.005	0
0.001	1164	0.018	515	0.022	0	0	0
0.002	0	0	63	0.003	650	0.002	0
0.002	110	0.002	1320	0.056	2502	0.009	0
<0.001	26	<0.001	0	0	4904	0.017	0
0	690	0.011	1248	0.053	0	0	0
0.001	198	0.003	0	0	0	0	0
0.005	159	0.002	0	0	920	0.003	445
0.014	0	0	0	0	0	0	0
0.013	0	0	0	0	0	0	0
0.006	0	0	0	0	788	0.003	0
0	0	0	0	0	3499	0.012	0
0	1091	0.017	0	0	0	0	0
<0.001	0	0	0	0	0	0	0
0	0	0	0	0	0	0	0

0	0	0	0	0	2874	0.01	0
0.011	0	0	84	0.004	0	0	0
0.001	446	0.007	0	0	0	0	0
0	0	0	0	0	264	0.001	0
0.004	0	0	139	0.006	1030	0.004	0
0	0	0	461	0.02	367	0.001	0
0	780	0.012	0	0	0	0	0
0.001	54	0.001	0	0	47	<0.001	0
<0.001	0	0	0	0	259	0.001	0
0.008	0	0	0	0	0	0	0
<0.001	247	0.004	281	0.012	0	0	0
0.006	0	0	0	0	0	0	0
0	605	0.009	0	0	0	0	0
0	805	0.012	0	0	0	0	0
0.006	0	0	0	0	0	0	0
0.006	0	0	0	0	0	0	0
0.002	0	0	0	0	793	0.003	0
0.006	0	0	0	0	0	0	0
0	16	<0.001	703	0.03	0	0	0
0.004	0	0	0	0	0	0	0
0.002	42	0.001	0	0	205	0.001	0
<0.001	60	0.001	0	0	123	<0.001	108
0	0	0	0	0	0	0	0
0	304	0.005	213	0.009	0	0	0
0	0	0	0	0	274	0.001	0
0	87	0.001	0	0	0	0	0
0	289	0.004	205	0.009	0	0	0
<0.001	0	0	560	0.024	0	0	0
0.001	38	0.001	0	0	42	<0.001	0
0.001	0	0	38	0.002	338	0.001	0
0.001	0	0	0	0	130	<0.001	0
0	0	0	0	0	0	0	0
0.002	0	0	0	0	0	0	0
0	0	0	0	0	544	0.002	0
0	0	0	0	0	0	0	0
0	0	0	0	0	0	0	0
0.002	0	0	0	0	0	0	0
0	0	0	0	0	0	0	0
0.002	0	0	0	0	0	0	0
0.001	0	0	0	0	64	<0.001	0
0	0	0	0	0	53	<0.001	0
0.001	0	0	0	0	0	0	0
0	191	0.003	0	0	0	0	0
0.002	0	0	0	0	0	0	0
<0.001	0	0	0	0	256	0.001	0
0	161	0.002	0	0	0	0	0
0	0	0	0	0	0	0	0
0.001	0	0	0	0	0	0	0
0.001	0	0	0	0	0	0	0
0	26	<0.001	39	0.002	0	0	0
0	0	0	0	0	0	0	0
0	0	0	0	0	0	0	0
0	88	0.001	0	0	0	0	0
0.001	0	0	0	0	0	0	0
<0.001	0	0	0	0	89	<0.001	0
<0.001	0	0	0	0	0	0	0
0.001	0	0	0	0	0	0	0
<0.001	0	0	95	0.004	0	0	0
0	62	0.001	0	0	0	0	0
0	0	0	0	0	75	<0.001	0
0.001	0	0	0	0	0	0	0
0	0	0	0	0	0	0	0

<0.001	0	0	0	0	0	0	0
<0.001	0	0	0	0	0	0	0
<0.001	0	0	0	0	64	<0.001	0
<0.001	0	0	0	0	0	0	0
0	0	0	0	0	0	0	0
<0.001	0	0	0	0	0	0	0
0	0	0	0	0	0	0	0
<0.001	0	0	0	0	0	0	0
<0.001	0	0	0	0	0	0	0
<0.001	0	0	0	0	0	0	0
0	0	0	0	0	75	<0.001	0
<0.001	0	0	0	0	0	0	0
0	0	0	0	0	0	0	0
0	12	<0.001	0	0	0	0	0
<0.001	0	0	0	0	0	0	0
<0.001	0	0	0	0	0	0	0
<0.001	0	0	0	0	0	0	0
<0.001	0	0	0	0	0	0	0
<0.001	0	0	0	0	0	0	0
<0.001	0	0	0	0	0	0	0
<0.001	0	0	0	0	0	0	0
0	35	0.001	0	0	0	0	0
0	0	0	0	0	0	0	0
<0.001	0	0	0	0	0	0	0
<0.001	0	0	0	0	0	0	0
0	0	0	0	0	0	0	0
0	0	0	0	0	0	0	0
0	15	<0.001	0	0	0	0	0

nt #8	Patient #9		Patient #10	
Abundance (%)	Reads	Abundance (%)	Reads	Abundance (%)
59.965	43466	0.115	846096	3.605
28.895	25859912	68.3	17053253	72.662
0.004	3256949	8.602	1005127	4.283
0.017	5112243	13.502	837287	3.568
0.002	1351	0.004	676	0.003
2.919	22731	0.06	0	0
0	20759	0.055	9977	0.043
<0.001	149818	0.396	53037	0.226
0.004	281919	0.745	10688	0.046
1.091	136298	0.36	234871	1.001
0.001	39764	0.105	19414	0.083
0.929	898	0.002	25711	0.11
0.001	109	<0.001	0	0
0.214	69111	0.183	70316	0.3
0	0	0	38639	0.165
0	2403	0.006	71	<0.001
0	0	0	0	0
0.026	24381	0.064	7346	0.031
0.076	25664	0.068	4081	0.017
0.126	8623	0.023	170	0.001
<0.001	13696	0.036	22448	0.096
0	15008	0.04	18405	0.078
0.002	464	0.001	713	0.003
0.06	192	0.001	1438	0.006
0	0	0	0	0
0.176	2841	0.008	0	0
0.001	5197	0.014	54	<0.001
0.037	1511	0.004	4667	0.02
0.058	3115	0.008	3631	0.015
0	0	0	6730	0.029
<0.001	660	0.002	121	0.001
0.007	0	0	1883	0.008
0	0	0	0	0
0	0	0	0	0
0	0	0	1689	0.007
0.042	3274	0.009	1576	0.007
0	5290	0.014	6516	0.028
0	0	0	0	0
0	154	<0.001	0	0
0	0	0	2516	0.011
0	0	0	0	0
0	0	0	0	0
0	0	0	0	0
0	0	0	331	0.001
0	0	0	0	0
0	0	0	1090	0.005
0	0	0	389	0.002
0	230	0.001	0	0
0	0	0	0	0
0	0	0	1879	0.008
0	0	0	0	0
0.002	0	0	408	0.002
0	0	0	0	0
0	0	0	0	0
0	498	0.001	157	0.001
0	0	0	0	0
0	0	0	0	0
0	0	0	0	0
0	132	<0.001	0	0

0	0	0	0	0
0	0	0	116	<0.001
0	0	0	1047	0.004
0	351	0.001	0	0
0	0	0	61	<0.001
0	0	0	0	0
0	0	0	0	0
0	0	0	354	0.002
0	0	0	0	0
0	0	0	0	0
0	257	0.001	199	0.001
0	0	0	0	0
0	0	0	0	0
0	0	0	0	0
0	0	0	0	0
0	0	0	0	0
0	0	0	0	0
0	0	0	0	0
0	0	0	0	0
0	0	0	0	0
0	144	<0.001	46	<0.001
0.001	519	0.001	0	0
0	0	0	0	0
0	0	0	0	0
0	0	0	588	0.003
0	0	0	0	0
0	0	0	0	0
0	0	0	0	0
0	0	0	59	<0.001
0	0	0	59	<0.001
0	0	0	132	0.001
0	49	<0.001	0	0
0	0	0	0	0
0	0	0	0	0
0	0	0	0	0
0	0	0	0	0
0	0	0	0	0
0	0	0	0	0
0	0	0	0	0
0	0	0	0	0
0	0	0	0	0
0	0	0	0	0
0	0	0	0	0
0	0	0	0	0
0	0	0	0	0
0	0	0	0	0
0	0	0	0	0
0	0	0	0	0
0	0	0	0	0
0	72	<0.001	0	0
0	0	0	0	0
0	0	0	0	0
0	0	0	0	0
0	0	0	0	0
0	0	0	0	0
0	0	0	0	0
0	0	0	0	0
0	0	0	0	0
0	0	0	0	0
0	0	0	0	0
0	0	0	0	0
0	0	0	0	0
